# Vertically transferred maternal immune cells promote neonatal immunity against early life infections

**DOI:** 10.1038/s41467-021-24719-z

**Published:** 2021-08-04

**Authors:** Ina Annelies Stelzer, Christopher Urbschat, Steven Schepanski, Kristin Thiele, Ioanna Triviai, Agnes Wieczorek, Malik Alawi, Denise Ohnezeit, Julian Kottlau, Jiabin Huang, Nicole Fischer, Hans-Willi Mittrücker, Maria Emilia Solano, Boris Fehse, Anke Diemert, Felix R. Stahl, Petra Clara Arck

**Affiliations:** 1 Division of Experimental Feto-Maternal Medicine, Department of Obstetrics and Fetal Medicine, University Medical Center Hamburg, Hamburg, Germany; 2 Developmental Neurophysiology, Center for Molecular Neurobiology Hamburg, University Medical Center Hamburg-Eppendorf, Hamburg, Germany; 3 Department of Stem Cell Transplantation, University Medical Center Hamburg-Eppendorf, Hamburg, Germany; 4 Bioinformatics Core, University Medical Center Hamburg-Eppendorf, Hamburg, Germany; 5 Institute for Medical Microbiology, Virology and Hygiene, University Medical Center Hamburg-Eppendorf, Hamburg, Germany; 6 Institute for Clinical Chemistry and Laboratory Medicine, University Medical Center Hamburg-Eppendorf, Hamburg, Germany; 7 Department of Immunology, University Medical Center Hamburg-Eppendorf, Hamburg, Germany; 8 Present Address: Department of Anesthesiology, Perioperative and Pain Medicine, Stanford University, Palo Alto, California, CA USA

**Keywords:** Differentiation, Epigenetic memory, Haematopoiesis, Reprogramming, Haematopoiesis

## Abstract

During mammalian pregnancy, immune cells are vertically transferred from mother to fetus. The functional role of these maternal microchimeric cells (MMc) in the offspring is mostly unknown. Here we show a mouse model in which MMc numbers are either normal or low, which enables functional assessment of MMc. We report a functional role of MMc in promoting fetal immune development. MMc induces preferential differentiation of hematopoietic stem cells in fetal bone marrow towards monocytes within the myeloid compartment. Neonatal mice with higher numbers of MMc and monocytes show enhanced resilience against cytomegalovirus infection. Similarly, higher numbers of MMc in human cord blood are linked to a lower number of respiratory infections during the first year of life. Our data highlight the importance of MMc in promoting fetal immune development, potentially averting the threats caused by early life exposure to pathogens.

## Abstract (web summary)

Maternal immune cells seed into the foetus during mammalian pregnancy, yet the functional role of these cells is unclear. Here the authors show that maternal immune cells in foetal bone marrow stimulate immune development, subsequently reducing the risk or severity of infections in newborns.

## Introduction

Fetal growth and development are greatly dependent on the mother. The placenta as the interface between mother and fetus is crucial in supporting fetal development and early life immunity, i.e., via the vertical transfer of growth factors, nutrients, oxygen, and pathogen-specific antibodies. Moreover, immune cells are vertically transferred from the maternal circulation to the fetus. These maternal cells then occur at very low frequencies in offspring’s organs; therefore, they are referred to as maternal microchimeric cells (MMc)^1–3^. Across mammalian species, MMc can be detected in a variety of fetal, neonatal, and even adult organs, including lymphoid tissues^4^.

To date, it is still debated whether MMc play a functional role in the offspring, or are simply accidental placental spillovers^2^. It has been hypothesized that MMc convey health consequences for the offspring. Evidence published to date suggests advantageous effects, such as the promotion of immune tolerance and the correction of shortcomings of the offspring’s immune system^5–10^. For example, in mouse offspring genetically deficient for distinct immune markers, MMc have been described to substitute this shortage, e.g., by secreting IgG in B-cell-deficient^7^ or IL-2 in IL-2-deficient offspring^10^. Translational relevance of these findings has been confirmed, e.g., in a neonate lacking mature T cells due to X-linked severe combined immunodeficiency (SCID-Xl) syndrome. Here, maternal T cells massively expanded when the child suffered from a viral infection caused by Epstein–Barr virus, which can be fatal in SCID-Xl patients^9^. Conversely, potentially disadvantageous consequences have been associated with MMc, including graft-versus-host like reactions or autoimmune diseases^11–16^ (reviewed in refs. ^2,4^). The ambiguity between these observations underpins the need for additional research aiming to dissect a functional role of MMc for offspring’s immunity.

The immune system largely develops during fetal life in humans and mice^2,17^. The re-location of fetal hematopoiesis from the liver to the bone marrow during late mouse gestation constitutes a hallmark of immune ontogeny. Here, hematopoietic stem cells (HSC) give rise to all myeloid and lymphoid blood lineages for the remainder of the offspring’s life^18^. HSC can self-renew for life and evidence suggests that DNA methylation directly regulates self-renewal and commitment to lymphoid and myeloid lineages. Myeloid and lymphoid commitment of HSC is epigenetically regulated and a comprehensive map of their methylome has been described^19^. Interestingly, the epigenome is particularly prone to challenges during development due to extensive DNA synthesis^20^.

Strikingly, the timing of vertical MMc transfer aligns with fetal immune ontogeny. Since MMc can be detected in the fetal immune organs, such as the bone marrow and thymus^4^, we here primarily investigated whether MMc modulate hematopoiesis in fetal bone marrow. We further tested if MMc-induced modulation of fetal immune development leads to beneficial or detrimental consequences for offspring’s immunity later in life.

The neonate is relatively well equipped to mount early life immunity against pathogen challenges, while averting overshooting immune activation against the initial colonization with commensal microbiota^21–23^. However, early life immunity is fragile and neonates are prone to infections, which creates a major threat to offspring’s survival and future health^24,25^. It is unknown if and how such increased risk for infections is triggered by an altered frequency or phenotype of MMc.

The identification of maternal microchimerism as a prerequisite to study their function long relied on DNA-based techniques, targeting genetic markers discordant between mother and child^2,4^. These approaches greatly restricted the possibility to decipher the phenotype of MMc. Moreover, the lack of tools to selectively manipulate MMc within fetal tissues has further precluded the identification of their functional role.

In this work, we have established mouse models in which the frequency of MMc can be altered before birth. We utilize preclinical models and advanced cytometry techniques to characterize and modulate MMc in mice in order to identify their physiological role in modulating fetal immune development and early life immunity. We show that MMc promote fetal immune development and improve neonatal immunity in mice, revealed by a reduced risk for early life viral infections. The translational relevance of our findings in mice is supported by our observation that higher number of MMc in neonatal cord blood are associated with a lower risk for respiratory infections in infants.

## Results

### Maternal microchimeric cells are present at important sites of the immune system in fetal and adult offspring

**Fig. 1:**
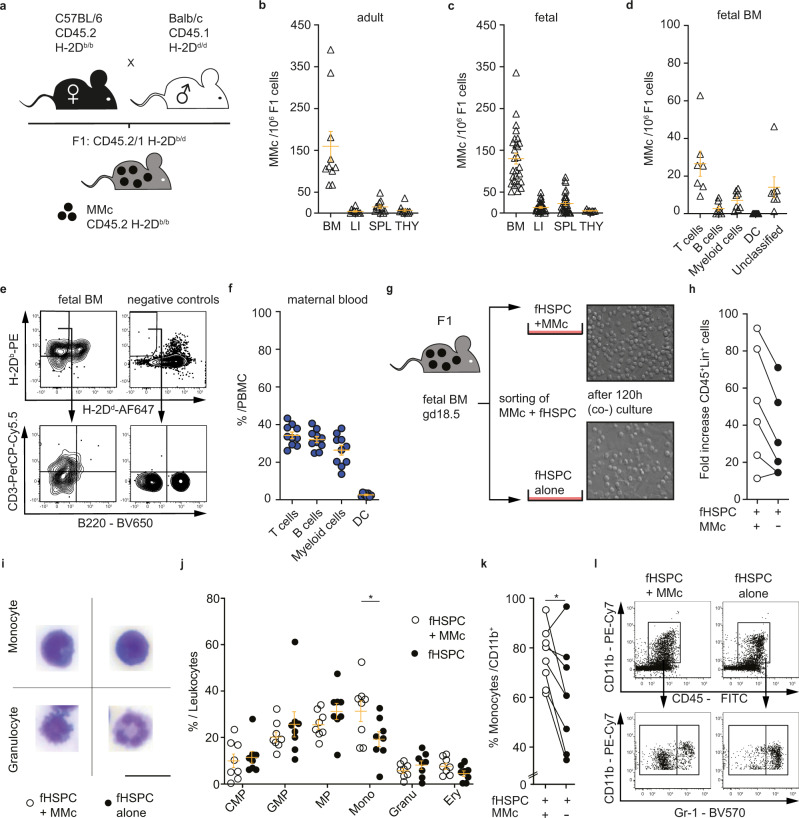
The majority of MMc in the fetal bone marrow are CD3^+^ T cells. **a** Mating strategy to identify maternal microchimerism (MMc) in offspring of Balb/c-mated C57BL/6 females by flow cytometry. MMc are identified in a two-step gating strategy for both maternal CD45.2 *and* H-2D^b/b^ cells among fetal CD45.2/1^+^H-2D^b/d^ cells. **b** Number of MMc /1 × 10^6^ cells in organs taken from adult offspring at 10–14 weeks of age (bone marrow (BM) *n* = 10; liver (LI) *n* = 10; spleen (SPL) *n* = 11; thymus (THY) *n* = 11), and **c** number of MMc /1 × 10^6^ fetal cells detected in fetal (F1) organs on E18.5 (BM *n* = 27; LI *n* = 28; SPL *n* = 28; THY pooled from *n* = 8 litters). **d** Frequencies of CD3^+^ T cells, CD3^neg^CD11b^neg^B220^+^ B cells, CD3^neg^CD11b^+^ myeloid cells, CD11c^+^ dendritic cells (DC) among magnetic cell sorting-enriched MMc in fetal BM and the subset that could not be classified by the panel used (E18.5, *n* = 14, each triangle represents a sample pooled from two fetuses (3 litters)). **e** Representative dot plot of maternal microchimeric cells (CD45.2^+^ and H-2D^b/b^) among fetal CD45.2/1^+^H-2D^b/d^ cells, and negative control (fluorescence-minus-one (FMO)) staining for H-2D^b^ (top right), and CD3 (bottom right). **f** Frequencies of subsets shown in **d** in the potential origin of MMc, the maternal peripheral blood (*n* = 8 mothers). **g** Workflow of co-culturing MMc and fetal hematopoietic stem and progenitor cells (fHSPC) (Lin^neg^Sca-1^+^c-Kit^+^), both isolated by fluorescence-activated cell sorting (FACS) from fetal bone marrow on E18.5 and pooled per litter, compared to culture of fHSPC alone. Top photomicrograph of fHSPC-derived cells after co-culture shows a higher cell number after culture in the presence of MMc, compared to fHSPC culture alone (bottom) (bar = 100 µm, magnification: 20x). **h** Number of CD45^+^Lin^+^ cells differentiating from fHSPC upon co-culture, as determined by flow cytometry. Depicted is the fold increase of CD45^+^Lin^+^ cell counts after 120 h of culture over the number of fHSPC initially seeded on day 0 of culture (*p* = 0.06, cells sorted from *n* = 6 independent litters examined in 6 independent experiments). **i** Representative cytospins (bar = 30 µm). **j** Frequencies of subset populations among cells derived from fHSPC in culture, evaluated by cell morphology after cytospin (methods). Eight independent co-culture experiments were performed. **k** Frequencies of Gr-1^lo/neg^ monocytes among CD11b^+^CD45^+^ myeloid cells derived from fHSPC in culture, evaluated by flow cytometry (*p* = 0.039, cells sorted from *n* = 8 independent litters examined in 8 independent experiments) and (**l**) representative dot plots. Scatter-plots in **b**–**d**, **f**, **j** represent mean ± SEM, **h** each pair of dots represents one fHSPC sample, cultured with or without MMc. **j** Mann–Whitney-U Test, two-sided, fHSPC+MMc vs. fHSPC alone, *p* = 0.037; **h**, **l** Wilcoxon-Rank-Sum Test, two-sided. Abbreviations not introduced in text: CMP common-myeloid progenitor, GMP granulocyte-monocyte progenitor, MP megakaryocyte-erythrocyte progenitor, Mono monocyte, Gran granulocyte, Ery erythrocyte. See also Supplementary Figs. 1 and 2. Source data are provided as a Source Data file.

We utilized an established allogeneic mating model in mice to identify MMc in offspring’s organs. MMc were identified by flow cytometry in a two-step gating strategy, based on homozygosity for CD45.2 among CD45.1/2 fetal cells and MHC class I H-2D^b/b^ among H-2D^b/d^ fetal cells. This allows for the detection of MMc among fetal host cells (CD45.2/1 H-2D^b/d^) (Fig. 1a). The limit of detection for this approach has been previously determined and is similar to MMc identification using quantitative real-time polymerase chain reaction (qRT-PCR)^26,27^. We show that MMc are present in adult tissues and can also be detected in fetal immune organs at late gestation (E18.5) (Fig. 1b, c). In fetal and adult offspring alike, highest MMc numbers can be found in the bone marrow.

### The fetal bone marrow is enriched in MMc T cells

In order to identify the phenotype of MMc, we focused on the site of highest frequency, the fetal bone marrow late in gestation. Aiming to overcome the technical challenges related to phenotypic assessments within small cell subsets, fetal bone marrow-derived MMc subsets were enriched by magnetic-activated cell sorting (MACS) prior to flow cytometry analyses (Supplementary Fig. 1a). This yielded to a 32.3 times higher frequency of MMc among fetal leukocytes in bone marrow, compared to non-enriched MMc assessments (mean frequency of MMc in enriched fraction was 4.2%, compared to 0.13% in non-enriched ex vivo bone marrow cell suspensions) (Supplementary Fig. 1b). The major cellular subsets within the MMc population could be identified as T cells, followed by myeloid cells, B cells, and dendritic cells (Fig. 1d, e). Some of the CD45^+^ MMc could not be classified using the major immune cell lineage markers we selected. Since maternal blood is considered to be the origin of vertically transferred MMc, we also assessed the relative distribution of T, B, and myeloid MMc cell subsets in peripheral blood of the mothers and found it to be clearly distinct from the composition of MMc cell subsets detected in fetal bone marrow (Fig. 1f), suggesting a selective vertical transfer and enrichment of T cells in the bone marrow. We injected some mothers i.v. with APC-Cy7-conjugated anti-CD45.2 shortly before culling. We did not detect APC-CD45^+^ cells in fetal blood or bone marrow. Also, fetuses did not show signs of visible maternal blood contamination upon preparation, which allows to exclude that maternal blood contamination during fetal tissue preparation may have confounded subsequent assessments of MMc in fetal organs (Supplementary Fig. 1c–e).

### MMc promote differentiation of fetal bone marrow hematopoietic stem and progenitor cells into myeloid cells in vitro

Intrigued by the selective enrichment of MMc in the fetal bone marrow, the primary site for hematopoiesis during late fetal development in mice, we tested if MMc modulate the differentiation of fetal hematopoietic stem and progenitor cells (fHSPC). We sorted fHSPC from the bone marrow of fetal offspring and cultured these cells in the presence or absence of MMc (isolated from the same host) on an embryonic bone marrow stromal cell line (OP9) feeder layer (Fig. 1g). Here, a preferential differentiation into CD45^+^Lineage (Lin)^+^ cells could be detected when fHSPC were cultured in the presence of MMc, compared to fHSPC cultured on the OP9 feeder layer alone (Fig. 1h). Confounding factors such as cell death or alterations in stromal cell frequencies in the absence or presence of MMc could be excluded (Supplementary Fig. 2a–c). These findings highlight that MMc support proliferation and differentiation of fHSPC into CD45^+^Lin^+^ cells in vitro. The OP9 feeder layer is devoid of markers promoting lymphoid differentiation. Hence fHSPC differentiation towards the myeloid lineage occurs by default. Despite these limited experimental boundary conditions, we observed a significantly higher differentiation of fHSPC into monocytes when cultured in the presence of MMc, compared to in vitro culture of fHSPC on OP9 feeder cells alone, as determined by morphological phenotyping. Other progenitor and terminally differentiated populations remained unchanged under the respective culture conditions (Fig. 1I, j; Supplementary Fig. 2d–f). Our findings from the morphological assessments were independently confirmed using flow cytometry. Here, the preferential differentiation of fHSPC into monocytes (defined as CD11b^+^Gr-1^low/neg^) could also be detected upon fHSPC culture in the presence of MMc (Fig. 1k, l). Taken together, our observations strongly support that MMc promote the proliferation of fHSPC and, within the myeloid compartment, favor their differentiation into monocytes in vitro. Since this in vitro approach has limitations, e.g., the difficulty to use suitable mock cells in lieu of MMc, we next aimed to test the functional role of MMc in in vivo settings.

### Establishment of a mouse model to assess the functional role of MMc n vivo

**Fig. 2:**
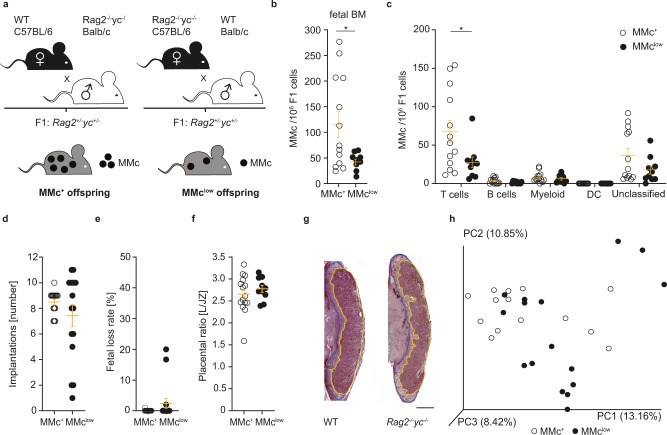
Development of a model with reduced MMc transfer from mother to fetus. **a** Mating strategy to produce offspring with reduced number of maternal microchimeric cells (MMc) (MMc^low^), resulting from the T, B cell and natural killer (NK) cell immunodeficiency in *Rag2*^-/-^γc^-/-^ mice^28^, which we reciprocally used as mothers or fathers. Offspring from wild-type (WT) C57BL/6 females were termed MMc^+^, and from *Rag2*^-/-^γc^-/-^ C57BL/6 females, MMc^low^. **b** Number of MMc /1 × 10^6^ fetal cells in fetal bone marrow on E18.5 in MMc^low^ offspring in comparison to number of MMc /1 × 10^6^ fetal cells in MMc^+^ control fetuses (MMc^+^ *n* = 26 (6 litters), MMc^low^ *n* = 16 (4 litters), each dot represents a pooled sample of two fetuses (*p* = 0.0267). MMc are identified in a two-step gating strategy for both maternal CD45.2 and H-2D^b/b^ cells among fetal CD45.2/1^+^H-2^b/d^ cells. **c** Frequencies of CD3^+^ T cells (*p* = 0.0303), CD3^neg^CD11b^neg^B220^+^ B cells, CD3^neg^CD11b^+^ myeloid cells, CD11c^+^ dendritic cells (DC) among magnetic cell sorting-enriched MMc in fetal BM of MMc^+^ and MMc^low^ offspring and the subset that could not be classified by the panel used (E18.5, female offspring only, for *n* see **b**. **d**–**g** Assessment of the reproductive outcome of matings depicted in **a**. **d** shows the number of implantations on E18.5, **e** fetal loss rate, **f** ratio of placental labyrinth (L) to the junctional zone (JZ) (L/JZ ratio) as a proxy for placental function on E18.5 (MMc^+^ placentae from 8 litters, MMc^low^ placentae from 8 litters). **g** Representative photomicrographs of placentae stained using Masson’s Trichrome Staining to distinguish L (circled in yellow) from JZ (circled in blue). Bar = 1000 µm. **h** Phylogenetic distances between intestinal microbiome samples, calculated via weighted UniFrac distance matrix. Each dot in the scatter plot represents a microbiome sample. The percentage of variation explained by each principal component (PC) is indicated on the axes. MMc^+^ (*n* = 16) and MMc^low^ (*n* = 13) offspring (F1) on postnatal day 8 do not significantly differ with regard to their weighted UniFrac distance based on ANOSIM. Scatter-plots in **b** represent ± SEM, Student’s *t* test, two-sided. **c**–**f** Scatter-plots represent mean ± SEM, Mann–Whitney-U Test, two-sided. Statistics on **h** are described in the methods. See also Supplementary Fig. 3. Source data are provided as a Source Data file.

In order to assess the functional role of MMc during fetal hematopoiesis in vivo, we developed a mouse model in which the offspring are significantly devoid of MMc. Since we had identified the majority of MMc to be T and B cells, we utilized *Rag2*^−/−^γ*c*^−/−^ female mice as mothers, which are deficient for T cell, B cell and NK cells^28^. *Rag2*^+/−^γc^+/−^ offspring from *Rag2*^−/−^γ*c*^−/−^ C57BL/6 females allogeneically mated to wild-type Balb/c males were termed MMc^low^. Vice versa, *Rag2*^+/−^γc^+/−^ offspring arising from the reciprocal mating combination of wild-type C57BL/6 females to *Rag2*^-/-^γ*c*^-/^ Balb/c males were termed MMc^+^ (Fig. 2a). Noteworthy, the common γ chain (γc) gene is encoded by the X-chromosome and hence, male offspring born to *Rag2*^−/−^γc^−/−^ females (here termed MMc^low^) are γc deficient, while male offspring born to wild-type females (termed MMc^+^) carry one copy of the γc gene. To control for this hemizygosity, only female offspring were included in the respective experiments. As a proof of concept, we observed a significant reduction of MMc in MMc^low^ offspring, compared to the number of MMc detectable in MMc^+^ offspring at E18.5 (Fig. 2b). Indeed, a reduction of T cells and—albeit very low in numbers—also B cells primarily accounted for the lower MMc levels in bone marrow of MMc^low^ fetuses. Noteworthy, we observed some T cells among the MMc in MMc^low^ offspring. Since *Rag2*^−/−^γ*c*^−^ mothers do not have any T cells to transfer into the fetuses, the observed putative maternal T cells reflect the sensitivity limit of the assay. Within the MMc population, we detected myeloid and dendritic cells, as well as a cell subset which could not be classified using the markers we chose. The presence of these cell subsets within the MMc population was anticipated even in offspring from *Rag2*^−/−^γc^−/−^ females and indeed, the abundance of these non-T-cell populations was not significantly different between MMc^+^ and MMc^low^ fetuses (Fig. 2c). Similar to the wild-type model, MMc and subsets were identified upon MACS-based enrichment in the MMc^+^ / MMc^low^ model.

Prior to further assessments of MMc-related effects on HSC and offspring’s immunity in MMc^+^ and MMc^low^ offspring, we excluded potential confounders that may affect the designated read out parameters. Here, we did not observe significant differences affecting the reproductive outcome and fetal development between the mating combinations leading to MMc^+^ and MMc^low^ offspring, including gestational weight gain, plug to pregnancy rate, maternal gestational cytokine levels (TNF, IFN-γ, MCP-1, IL-6), number of fetal implantations, fetal weight, and loss rate or placental features (Fig. 2d–g; Supplementary Fig. 3a–f). Moreover, since the composition of the maternal microbiome can influence offspring’s microbiome and innate immune development^29,30^, we analyzed the gut microbiome of *Rag2*^−/−^γ*c*^−/−^ and wild-type mothers and their respective MMc^+^ and MMc^low^ offspring. Using full-length 16 S rRNA amplicon sequencing, differences of intestinal microbiota composition were observed between *Rag2*^−/−^γ*c*^−/−^ and wild-type mothers, as expected from published data^31^. However, no significant differences of the microbiome were present between neonatal MMc^+^ and MMc^low^ offspring (Supplementary Fig. 3g–i). Since the significant differences of gut microbiota between adult and neonatal mice of the respective groups may camouflage differences within the F1 subgroups in this analysis, we also assessed the PCA restricted to the F1 samples and could confirm that the gut microbiota did not significantly differ between the two offspring groups (Fig. 2h). Also, a parental-specific inheritance of gene expression patterns could be ruled out, since expression of *Rag2* and γ*c* did not differ between cells isolated from fetal bone marrow of MMc^+^ and MMc^low^ offspring, while expression in both, MMc^+^ and MMc^low^ offspring, was significantly reduced compared to that in the wild-type offspring, as expected (Supplementary Fig. 3j–l). Lastly, we did not observe morphological differences of the hematopoietic niches of the bone marrow between MMc^+^ and MMc^low^ offspring at E18.5 (Supplementary Fig. 3m).

### MMc-promoted generation of monocytes in vivo

**Fig. 3:**
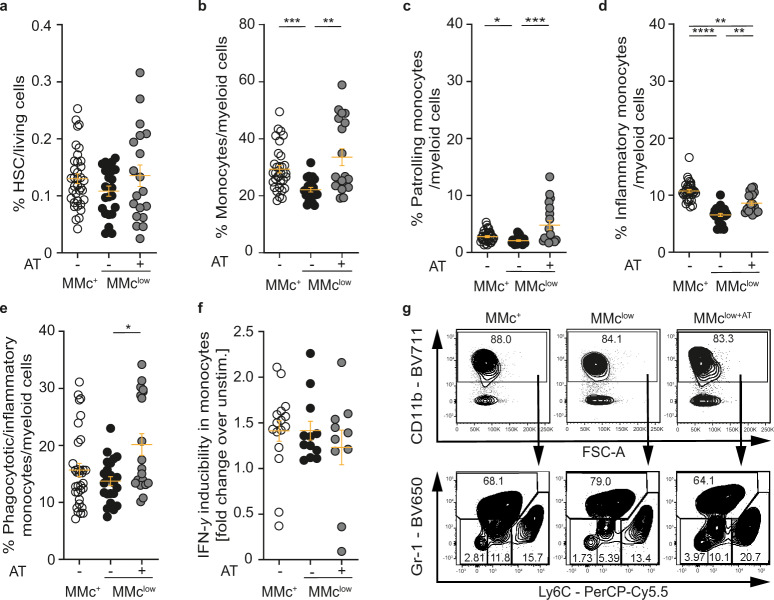
Rescued monocyte subset frequencies in MMc^low^ offspring by restoring MMc transfer. A third group was introduced to the mating schemes described in Fig. 2a in order to restore MMc transfer during pregnancy in *Rag2*^*-/-*^γ*c*^*-/-*^ mothers by adoptive transfer (AT) of immune cells isolated from secondary lymphoid organs of pregnant wild-type (WT) donor mice on E12.5. Offspring of this group was termed MMc^low+AT^ (Supplementary Fig. 4a, b). **a** Frequencies of hematopoietic stem cells (HSC) (Lin^neg^Sca-1^+^c-Kit^+^) in the fetal bone marrow (BM) in female MMc^+^ (*n* = 36), MMc^low^ (*n* = 23), and MMc^low+AT^ (*n* = 19) offspring on E18.5. **b** Frequencies of monocytes (Gr-1^lo/neg^ among CD11b^+^ myeloid cells) in fetal BM in female MMc^+^ (*n* = 33), MMc^low^ (*n* = 23), and MMc^low+AT^ (*n* = 19) offspring on E18.5 (MMc^+^ vs. MMc^low^ *p* = 0.0006; MMc^low^ vs. MMc^low+AT^ *p* = 0.0014). **c**–**e** Frequencies of monocyte subsets among CD11b^+^ myeloid cells in female MMc^+^, MMc^low^, and MMc^low+AT^ offspring on E18.5: **c** patrolling (Gr-1^neg^, Ly6C^low/neg^ (MMc^+^ vs. MMc^low^ *p* = 0.0163; MMc^low^ vs. MMc^low+AT^ *p* = 0.0010)), **d** inflammatory (Gr-1^neg^Ly6C^int^ (MMc^+^ vs. MMc^low^ *p* = <0.001; MMc^+^ vs. MMc^low^ *p* = 0.0050 MMc^low^ vs. MMc^low+AT^ *p* = 0.0067)), and **e** phagocytic/inflammatory (Gr-1^lo/neg^Ly6C^high^ (MMc^low^ vs. MMc^low+AT^ *p* = 0.0442) monocytes. **c**–**e** Number of female offspring per group: MMc^+^ *n* = 33 (9 litters), MMc^low^ *n* = 24 (6), MMc ^low+AT^ *n* = 19 (5). **f** IFN-γ production by monocytes in vitro (fold change refers to IFN-γ production after 4 h of stimulation with Phorbol myristate acetate (PMA)/ionomycin in cells isolated from BM of MMc^+^, MMc^low^, and MMc^low+AT^ offspring on E18.5 (same *n* as in **c**–**e**). **g** Representative dot-plots of Gr-1^lo/neg^ monocytes and their subsets (bottom row) and their parent population, CD11b^+^ myeloid cells (top row). CD11b^+^ myeloid cell frequencies among living singlet leukocytes were not different between groups (see also Supplementary Fig. 4c). **a**–**f** Scatter-plots represent mean ± SEM. Kruskal–Wallis test, Dunn test for post-hoc analysis, two-sided. Gating strategy is provided in Supplementary Fig. 4d. See also Supplementary Figs. 4 and 5. Source data are provided as a Source Data file.

Having ruled out major confounders which may affect hematopoiesis in the bone marrow in MMc^+^ and MMc^low^ offspring, and given the MMc-dependent preferential differentiation of fHPSC into nonocytic cells in vitro, we next assessed myeloid differentiation pathways in vivo. Here, we amended our model of generating MMc^+^ and MMc^low^ offspring by a third group, in which *Rag2*^−/−^γ*c*^−/−^ mothers received an adoptive transfer of syngeneic immune cells from a gestational age-matched WT donor female at mid-gestation (day 12.5), aiming to reconstitute MMc in MMc^low^ offspring (Supplementary Fig. 4a). Indeed, this adoptive transfer significantly restored MMc numbers in fetal MMc^low^ offspring (termed MMc^low+AT^) to levels seen in MMc^+^ offspring (Supplementary Fig. 4b). The frequency of Lin^neg^ Sca-1^+^ c-Kit^+^ fHSPC in fetal bone marrow was not significantly different between MMc^+^, MMc^low^, and MMc^low+AT^ offspring (Fig. 3a, Supplementary Fig. 4), which ruled out that differences in cell differentiation between groups resulted from different fHSPC frequencies. Analyses of myeloid cells in these offspring independently supported our in vitro observations of a MMc cell-promoted generation of monocytes, as we detected a significantly lower frequency of monocytes among myeloid cells in bone marrow of MMc^low^ offspring, which was fully restored in MMc^low+AT^ offspring (Fig. 3b). The absolute number of monocytes in these three groups mirror these findings on frequencies, whilst total number of cells isolated from the bone marrow did not significantly differ between the three groups (Supplementary Fig. 4c, d). In-depth analyses of key monocyte subsets^32,33^ revealed that the reduction of monocytes in the bone marrow of MMc^low^ offspring largely affected inflammatory and patrolling monocytes, whereas phagocytic/inflammatory monocytes where only marginally affected (Fig. 3c–e, g, Supplementary Fig. 4). The proof of principle that the observed effects on monocytes were indeed related to MMc could be provided by our adoptive transfer experiments, which completely restored the low frequencies within the respective monocyte subsets in MMc^low+AT^ fetuses (Fig. 3c–e, g). However, upon unspecific stimulation of fetal bone marrow cells to induce IFN-γ production, monocytes from MMc^+^ and MMc^low^ fetuses showed no differences with regard to cytokine production (Fig. 3f).

Taken together, these observations indicate that lineage commitment, but not function of monocytes is modulated by MMc, at least locally in the bone marrow. In a compensatory manner, higher frequencies of granulocytes could be observed among myeloid cells in MMc^low^ offspring along with significant differences of common myeloid progenitor (CMP) and granulocyte–monocyte progenitor (GMP) (Supplementary Fig. 5a–c). This suggests that MMc promote the lineage commitment towards the generation of monocytes at the post-GMP level, resulting in a compensatory differentiation in favor of granulocytes when MMc are low. This is supported by our n vitro findings in the presence or absence of MMc, as described above. We here also observed higher frequencies of GMP when MMc are absent, whereby the increase of granulocytes in the absence of MMc under in vitro culture conditions was subtle. The latter may be explained by the reduced availability of essential growth factors to support the expansion of granulocytes in vitro.

Since we also observed a reduction of common lymphoid progenitors (CLP) in bone marrow of MMc^low^ fetuses (Supplementary Fig. 5d), we performed pilot experiments in order to assess maturation of thymocytes in MMc^+^ and MMc^low^ fetuses, including double-negative (DN) stages and CD3, CD4, and CD8 expression (Supplementary Fig. 5e–n). Here, the key observation is a reduced frequency of CD8^+^ T cells in the thymus of MMc^low^ fetuses, which could subsequently contribute to an impaired effector response to pathogen challenges.

### Neonatal mice with reduced levels of MMc show an increased severity of viral infection

**Fig. 4:**
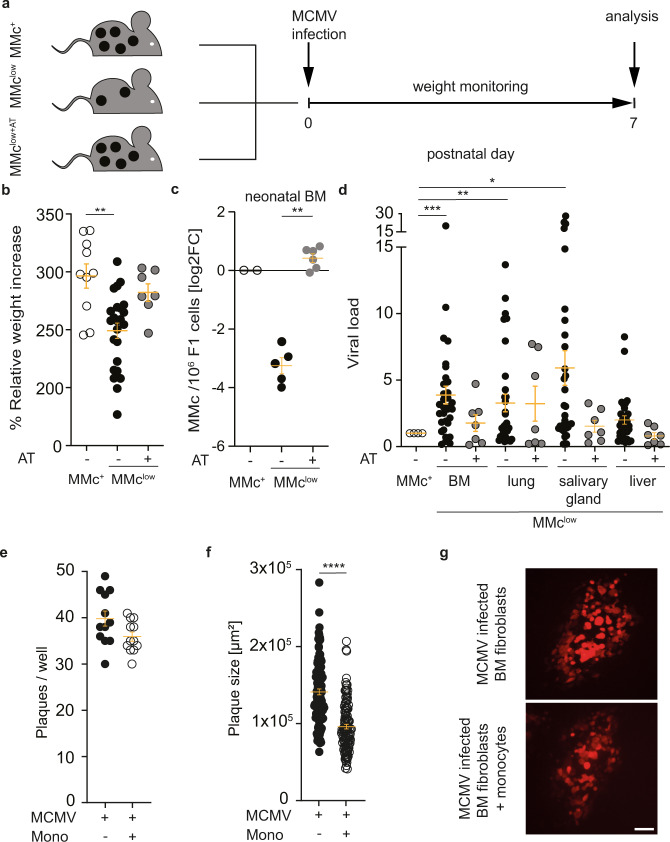
Increased severity of neonatal CMV infection in MMc^low^ offspring. **a** Experimental scheme of inoculating MMc^+^, MMc^low^, and MMc^low+AT^ offspring at day of birth with murine cytomegalovirus (MCMV, 3DR mutant, 1 × 10^4^ p.f.u. intratracheally) with subsequent weight monitoring, and analysis of viral load in organs 7 days post infection. **b** Weight increase on day 7 over birth weight as proxy for MCMV-related morbidity (MMc^+^ vs. MMc^low^ *p* = 0.0029), see **d** for *n*). **c** Number of MMc /1 × 10^6^ fetal cells in neonatal bone marrow (BM) on day 7 after birth in MMc^+^, MMc^low^, and MMc^low+AT^ at birth (non-infected female offspring only, depicted as log2foldchange (FC), MMc^+^ *n* = 2, MMc^low^ *n* = 5, and MMc^low+AT^ *n* = 6, (MMc^low^ vs. MMc^low+AT^ *p* = 0.0079). See also Supplementary Fig. 4b for fetal MMc levels in the three groups. **d** MCMV viral load in MMc^+^, MMc^low^, and MMc^low+AT^ offspring, determined as relative light units (RLU). Levels in MMc^low^ and MMc^low+AT^ are each normalized to levels in neonatal organs of infected MMc^+^ offspring (BM, lung, salivary gland, and liver). Data pooled from three independent experiments, each with similar result (infected: MMc^+^ *n* = 14 (6 litters), MMc^low^ *n* = 32 (9 litters) and MMc^low+AT^ *n* = 7 (3 litters); only female offspring/group; BM: MMc^+^ vs. MMc^low+AT^ *p* = 0.0009; lung: MMc^+^ vs. MMc^low^ *p* = 0.0059; salivary gland: MMc^+^ vs MMc^low^ *p* = 0.024. **e** Absolute numbers of plaques per well and **f** size per plaque (*p* = <0.0001). Pooled data obtained from four independent experiments. **g** Representative photomicrographs of MCMV plaques under the different culture conditions in the in vitro plaque reduction assay, confirmed by *n* = 4 independent experiments. MCMV (mCherry^+^) plaques were monitored using virus permissive bone marrow fibroblasts in the presence or absence of sorted monocytes (methods). **b**–**f** Scatter-plots represent mean ± SEM. **b**–**d** Kruskal–Wallis test, two-sided, Dunn test for post-hoc analysis. **e**, **f** Mann–Whitney test, two-sided. **g** 100 µm scale bar. See also Supplementary Fig. 5. Source data are provided as a Source Data file.

Monocytes are key early responders in infections and essential for the early control of infections^34,35^. Infection causes a pivotal health burden for neonates especially before vaccination regimen are completed. Infections induced by cytomegalovirus (CMV) can cause sensorineural hearing loss and severe neurological impairments. The severity of CMV infection is determined by the host’s immunity^36–38^. Therefore, infection of neonatal mice with the murine CMV (MCMV) has become an appreciated model to understand early life anti-CMV immunity^39^. By combing the neonatal MCMV infection model with the MMc^+^/MMc^low^/MMc^low+AT^ model (Fig. 4a), we next evaluated whether the reduced frequencies of distinct monocyte subsets and other features of altered immune cell subsets seen in MMc^low^ offspring may affect the course of neonatal MCMV infection. Indeed, infection of neonates with MCMV resulted in a higher morbidity in MMc^low^ neonates, as deduced from the significantly reduced neonatal weight gain between birth and postnatal day 7 (Fig. 4b). The severity of MCMV infection was mitigated upon restoring MMc, as seen in MMc^low+AT^ offspring (Fig. 4b). The numbers of MMc in bone marrow of infected neonates in the respective groups mirror the pattern observed in fetal mice (Fig. 4c). In MMc^low^ neonates, the significantly increased severity of MCMV infection was independently confirmed by increased MCMV viral titers in organs known to be affected by MCMV, such as the bone marrow, lung, salivary gland, and liver (Fig. 4d). Strikingly, this increased severity was largely restored in MMc^low+AT^ offspring (Fig. 4d). We could exclude that the protection from MCMV in neonates was mediated by vertical transfer of pathogen-specific antibodies, as MCMV titers were negative in mothers included in these experiments (Supplementary Fig. 5o). We did not test for a vertical neonate-to-mother MCMV transmission, which may have interfered with maternal nursing and caring and hence, differentially affected neonatal development in offspring nursed by WT vs. immunocompromised mothers. This omission is justified by the low amount of virus used to infect neonatal mice, and the fact that longitudinally recorded maternal weight and overall well-being of the mothers during the investigated neonatal infection period gave no indication for a maternal infection. Most importantly, the restoration of both weight increase and viral load to WT levels in infected MMc^low+AT^ neonates (born to and nursed by *Rag2*^−/−^γc^−/−^ mothers) supports that differences in maternal infection susceptibility and care are unlikely to having contributed to the observed infectious burden in MMc^low^ compared to WT offspring, yet further strengthens the hypothesis of neonate-intrinsic differential immunity. The direct involvement of neonatal monocytes in reducing the MCMV viral load was confirmed in vitro. Here, a significant reduction of the MCMV plaque sizes was detected in the presence of bone marrow-derived monocytes derived from wild-type neonates (Fig. 4e–g). It would have been desirable to also include monocytes isolated from MMc^+^ and MMc^low^ offspring, but the low number of monocytes in MMc^low^ offspring impeded the harvest of the required monocyte number for the in vitro MCMV plaque reduction assays. Moreover, we did not use different co-culture conditions with varying numbers of monocytes, as variations of cells within the required range for testing our hypothesis would have limited the validity of the assay. Together, our observation strengthens that neonatal monocytes interfere with MCMV dissemination. However, bone-marrow-derived monocyte-independent, yet MMc-modulated, pathways, such as an impaired T-cell response and possibly additional immune impairments may also contribute to the severe course of MCMV-infection observed in MMc^low^ neonates.

### MMc induce stable epigenetic alterations of the HSC methylome

Given that MMc modulate fetal immune development and affect postnatal immunity at multiple levels, including the generation of monocyte and T-cell subsets and response to MCMV infection, we next evaluated if MMc are capable of inducing epigenentic changes in HSC. We performed methylated DNA immunoprecipitation sequencing (MeDIP-Seq) on purified, bone marrow-derived HSC from MMc^low^ and MMc^+^ offspring (Supplementary Fig. 6a, Supplementary data 1). MeDIP-Seq allows for the detection of stable methylation changes, as opposed to assessments that are more rapidly reversible, such as histone modification. We analyzed HSC from adult offspring, which also facilitated to obtain the necessary number of HSC for MeDIP-Seq. We identified differentially methylated regions (DMR) between HSC genomes from MMc^low^ compared to MMc^+^ offspring mostly in distal intergenic regions, followed by promotor regions (Supplementary Fig. 6b, Supplementary data 2). Gene ontology analysis of affected biological process was performed using a cut-off (log2 fold change < -1.5 and >1.5, and FDR-adjusted *p*-value), which revealed that gene bodies and promoter regions involved in a vast range of biological functions were significantly hypo- and hypermethylated. (Supplementary Fig. 6c, Supplementary data 2).

### Maternal microchimerism in cord blood is inversely correlated with early life infections in human infants

**Fig. 5:**
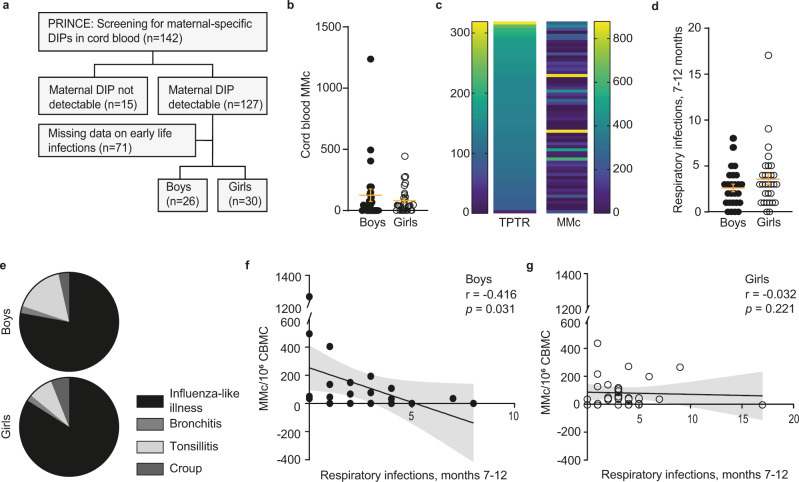
Cord blood MMc is inversely correlated with early life infections. **a** Flow chart for detection of maternal-specific DIPs in umbilical cord blood samples in a nested cohort derived from the PRINCE study (methods). Among 127 dyads in which a maternal-specific deletion–insertion polymorphisms (DIP) was detected, medical records on early life infections were available for 26 male and 30 female infants. **b** Absolute count of MMc among 10^6^ cord blood mononuclear cells (CBMC) at birth (26 boys, 30 girls). **c** Alignment of the transplacental IgG transfer rate (TPTR), calculated from IgG antibody levels against measles, mumps, rubella, tetanus, diphtheria, pertussis, and influenza A in maternal and cord blood, vs. number of MMc in cord blood of the same mother/neonate pairs. **d** Cumulative count of reported early life respiratory tract infection during months 7–12, stratified by infant sex (26 boys, 30 girls). **e** Sex-specific incidence of the distinct diseases subsumed as respiratory tract infections. **f**, **g** Correlation of early life respiratory tract infections in months 7–12 of life with the number of MMc in 10^6^ cord blood mononuclear cells (CBMC) at birth in boys (**f**), and girls (**g**). **b**, **d** Graphs represent mean ± SEM, Mann–Whintey-U test, two-sided. **f**, **g** Linear regression line (black), nonparametric Spearman correlation with a 95% confidence interval (shaded). *n* is represented in **a**. See also Supplementary Fig. 7. Source data are provided as a Source Data file.

We then aimed to test the translational relevance of MMc-dependent modulation of the risk for early life infections in humans. Here, we assessed maternal microchimerism at birth in children born within the PRINCE study by first screening for informative deletion and insertion polymorphisms (DIP) specific for the mother, which could be detected in 89.4% of the cases (Fig. 5a, Supplementary table 1). We subsequently used these DIPs to distinguish maternal from neonatal DNA in cord blood using a maternal DIP-specific duplex digital PCR^40^. Among the screened samples with informative maternal DIPs, the number of MMc (calculated from maternal DNA levels, but for simplicity referred to as MMc) was similar between boys and girls (Fig. 5b, Supplementary data 3). In a previous study using samples from the PRINCE study, we had tested IgG antibody levels against seven pathogens (measles, mumps, rubella, tetanus, diphtheria, pertussis, and influenza A) in maternal blood at gestation week 24 and in cord blood and calculated the transplacental IgG transfer rate (TPTR)^41^. We here aligned the number of MMc in cord blood to the TPTR and observed that vertical transfer of MMc occurs independent from IgG (Fig. 5c). Next, we assessed the number of early life respiratory infections (influenza-like illness, bronchitis, tonsillitis, croup, also known as laryngotracheobronchitis) during the second half of the first year of children’s life (months 7–12). Here, we observed no differences of total numbers or disease-specific incidences between boys and girls (Fig. 5d, e). We focused on this specific period in life, as neonates are no longer protected from infections by passive immunity resulting from the vertical transfer of maternal IgG, i.e., antibodies against influenza A^42^. Remarkably, we identified an association between MMc in cord blood and the subsequent onset of infections occurring during infancy at the age of 7–12 months, which reached levels of significance in boys (Fig. 5f), but not in girls (Fig. 5g). Despite the relatively low number of cases, we could perform multiple regression analysis^43^, which confirmed the significant impact of MMc in reducing the risk for early life infections especially in boys, compared to other known modulators of childhood infections, such as gestational age, birth weight, or the presence of older siblings (Supplementary Table 2). We previously observed that the level of antibodies against influenza A in cord blood reduces the risk for respiratory infections during the first 6 months of life^41^. Here, we re-evaluated these data in the context of MMc. Levels of antibodies against influenza did not differ between boys and girls and showed no correlation with MMc in cord blood in boys or girls (Supplementary Fig. 7a–c). This allows to exclude that our correlations were confounded by this serological marker, as also confirmed by the regression analysis (Supplementary Table 2). The types of infections also did not differ between boys and girls (Supplementary Fig. 7d, e). When correlating MMc in cord blood with respiratory infections over the entire first year of life (months 0–12), the inverse association seen for the period of 7–12 months was not present. This strongly suggests that MMc effects on infection risks during the first 6 month of life were camouflaged by the effect of antibodies. This notion further strengthens that MMc promote immunity and mitigate the risk for early life respiratory infections in human infants beyond the first 6 months of life, when neonatal protection by passive immunity has waned (Supplementary Fig. 7f, g).

## Discussion

We here provide evidence for an impact of MMc on fetal hematopoietic stem cells in mice, subsequently affecting neonatal immunity. MMc favor the differentiation of monocytes from fetal bone marrow-derived fHSPC and modulate T-cell development. Functionally, MMc enhance immunity towards early life pathogen challenges, as seen in an experimental model of early life infections, when neonatal mice were exposed to MCMV. Similarly, higher MMc in human neonates are linked to a lower number of respiratory infections in infancy. These findings underpin that vertically transferred maternal immune cells modulate fetal immune development and promote neonatal immunity. Since MMc can persist for a long period after birth, the maternally derived promotion of offspring’s immunity may exceed the antibody-mediated immunity in neonates, which wanes at an infant’s age of four to six months^29,42,44^.

In our experiments, we focused on the effect of MMc on immune cell differentiation in fetal bone marrow in mice, the primary site for hematopoiesis. Our data amend previous reports which describe maternal lymphocyte-like cells that are present in hematopoietic regions in fetal bone marrow cavities during late gestation^45–47^. Clearly, mouse hematopoiesis occurs under a distinct timeline during fetal development. Prior to the bone marrow, the fetal liver serves as a site for early hematopoiesis^48,49^ and further studies are needed to evaluate if the presence of MMc in the fetal liver at mid gestation in mice affects the expansion of hematopoietic progenitors and their subsequent maturation upon seeding in the bone marrow.

Previous work aiming to understand the possible effects of MMc focused on the modulation of fetal immune tolerance against non-inherited maternal antigens (NIMA) expressed by MMc, revealing the generation of offspring’s regulatory T cells with specificity to NIMA^5,6^. Although our present study did not focus on NIMA-mediated fetal tolerance, our experiments support these previous findings through the detection of allogeneic maternal cells, which are not being rejected until adulthood in mice.

The remarkable ability of MMc to modulate the differentiation of HSPC suggests that MMc may function as cellular shuttles, delivering cytokines and growth factors to the developing stromal niche and hematopoietic cells at a time when the fetus may not yet be capable of producing these factors itself. IL-2 has been suggested to be such a shuttled cytokine with regard to thymic function^10^. However, insights into distinct cytokines or growth factors that could fulfill such a function in the bone marrow are still unknown. Similarly, since bone marrow-derived progenitor cells seed into the fetal thymus during development and differentiate and mature into T cells^50,51^, functional assessments are now needed to prove if reduced or phenotypically altered MMc modulate the risk for T-cell-mediated diseases, such as autoimmunity and others^52^.

Myeloid cells in the neonatal lung localizing next to MCMV-infected cells in nodular inflammatory foci have been associated with clearance of the acute infection^39,53^. This is supported by our present work, where we could show that monocytes are directly involved in reducing the MCMV viral load in vitro. However, we could not observe significant changes of IFN-γ, a cytokine also produced by immature monocytes which reduces the risk for early life infection^54–57^, in fetal bone marrow-derived monocytes between groups. Here, we had stimulated fetal bone marrow bulk cells and not isolated monocytes, which may have camouflaged the induction of the monocyte-specific cytokine production. The difference in viral load after MCMV infection across organs encourages to investigate differential systemic anti-viral immunity between MMc^+^ and MMc^low^ neonates. Future work will address the kinetics of monocyte recruitment to the sites of infection and tissue-specific anti-viral responses, to better understand mechanisms behind the higher susceptibility of MMc^low^ neonates, and the impact of MMc on innate and adaptive immune development.

It has been observed that the maternal microbiome contributes to the early colonization of the offspring, which can then skew neonatal immunity^29^. In our study, we confirmed findings from others that the phylum *Bacteroides* dominates the intestinal microbiome in wild-type mice late in gestation^58^. In immunocompromised *Rag2*^−/−^γ*c*^−/−^ mothers, which gave birth to MMc^low^ offspring, shifts in the postpartal microbiome composition could be observed, as expected based on previous observations of differing microbiomes in immunodeficient (*Rag2*^*R229Q*^ knock-in) compared to WT males^31^. For example, the phylum *Verrucomicrobia* was found in immunocompromised *Rag2*^−/−^γ*c*^−/−^ mothers, but not in wild-type mice, suggesting that pregnancy uniquely alters the intestinal microbiome in Rag2^−/−^γc^−/−^ compared to wild-type mothers. However, since the neonatal intestinal microbiome did not significantly differ between MMc^+^ and MMc^low^ offspring, the impact of the maternal microbiome differences on the response to MCMV infection, which we used as a model system for early life immunity, seems to be negligible. We speculate that factors such as offspring’s genotype [which is the same in offspring from both mating combinations (*Rag*^*+/−*^γ*c*^*+/−*^)], and a shared housing environment shapes a similar microbial phenotype in these immune-competent offspring, at least at this cross-sectional time point 8 days after birth.

We focused on the assessment of a model in which the MMc reduction is achieved by using offspring from *Rag2*^−/−^γ*c*^−/−^ mother, resulting in a reduced vertical transfer of T and some B cells. However, our MMc phenotyping also shows that other leukocyte cell types such as myeloid cells are still present in MMc^+^ and MMc^low^ offspring, albeit levels did not significantly differ between groups. Hence, future studies including i.e., reciprocal matings of mice lacking CD11b^59^ would be useful to evaluate the functional role of MMc subsets other than T and B cells.

Besides the vertical transfer of maternal cells, maternally derived cytokines can also affect fetal development and long-term offspring’s health in a number of mammalian species. Prominent examples include the observation that the maternal interleukin-17a pathway in mice promotes autism-like phenotypes in offspring^60^. In humans, maternal IL-6 during pregnancy is linked to brain connectivity in newborns and predicts future working memory in offspring^61^. With regard to the risk for neonatal infections, it could be shown that newborn piglets are protected from Bordetella pertussis infection by a combination of passively transferred maternal cytokines and antibodies^62^. We did not observe differences between maternal cytokine levels at steady state in the MMc^+^/MMc^low^ model we here introduced. However, it should be noted that published evidence focuses on the vertical transfer of cytokines in the context of prenatal challenges and related pathological conditions, such as maternal immune activation triggered by adverse prenatal conditions due to infections or stress^63,64^. Considering the longevity of maternal cells compared to cytokine in offspring’s organs, future studies should aim at dissecting the effect of soluble, possibly rapidly inactivated maternal immune mediators such as cytokines—which may have a profound, but short-lived effect during fetal development—in the light of the long-term presence of maternal immune cells.

Our present findings amend the intriguing concept that maternal immune markers modulate offspring’s health and sex-specifically reduce the risk for early life infections in humans. One might argue that some of our findings may challenge existing data, i.e., as increased number of MMc have been observed in cord blood of neonates born prematurely^65^, whereby preterm born neonates are highly vulnerable to respiratory infections^66^. We here analyzed MMc and risk for infections early in life in term born children. Further, one must consider that time for vertical transmission of MMc is shorter in pregnancies affected by preterm birth, with yet unknown functional consequences. More importantly, preterm birth is often preceded by inflammation^66^, which may also affect the phenotype of MMc vertically transferred to the fetus, as shown in mice as well as in humans^65,67^.

Lastly, a number of findings presented here could only be obtained from female MMc^+/low^ offspring, as the γc gene is located on the X-chromosome, while the effect of MMc on human early life infections we here describe was greater in boys. This does not mean that MMc are irrelevant to reduce the risk for infections in girls. Rather, females are less prone to infections in the first place due to the fact that a number of genes important in sensing live virus or in initiating immune responses upon pathogen challenge are located on the X-chromosome and thus, can escape X-chromosome inactivation. These genes include TLR8, which is involved in Th1 differentiation, relevant in viral infections, or BTK (Bruton Tyrosine Kinase), involved in downstream signaling events of the B-cell receptor. Thus, we hypothesize that the threshold at which MMc significantly contribute to reducing the risk for infections in girls is higher compared to that in boys. This hypothesis should be tested in female infants with an inborn or imprinted higher risk for infections, e.g., in preterm children or upon prenatal challenges such as maternal smoking.

Taken together, our study provides strong evidence that MMc promote fetal immune development and enhance neonatal immunity, hereby mitigating the risk for early life infections. The effect of MMc on offspring’s immunity may be sustained into adulthood by stable epigenetic changes, which requires in-depth analysis in future research endeavors. Future endeavors now need to identify how to utilize these functions to promote MMc-mediated health advantages in neonates. MMc assessment may also be used as a predictive tool for immunity throughout life. Ultimately, the identification of additional functional roles of MMc outside immunity, for example in developmental processes like brain development, could be exploited to enhance children’s health and well-being.

## Methods

### Mice

Animal care and all experimental procedures were performed according to University Medical Center Hamburg-Eppendorf institutional guidelines and conform to requirements of the German Animal Welfare Act. Ethical approvals were obtained from the State Authority of Hamburg (Germany, approval numbers G10/067, G16/085, G17/010, G17/099, ORG_615, ORG_702, ORG_764, ORG_795).

C57BL/6 J (CD45.2, H-2D^b^), C57BL/6 *Rag2*^*−/−*^*Il2rg*^*−/*−^ (kindly provided by Dr. Peggy Riese, HZI Braunschweig), and Balb/c *Rag2*^*−/−*^*Il2rg*^*−/−*^ (kindly provided by Dr. Jan-Eric Turner, UKE Hamburg) mice were obtained from the animal breeding facility of University Medical Center Hamburg-Eppendorf. The *Il2rg* locus is referred to as γc (gamma chain) throughout the manuscript (*Rag2*^*−/−*^γ*c*^*−/−*^). Balb/c CD45.1 (CD45.1, H-2D^d^, CByJ.SJL(B6)-Ptprc^a^/J) were purchased from The Jackson Laboratory. Mice were single-housed (males) or maintained in groups (females) in the animal facility of University Medical Center Hamburg-Eppendorf with regular chow and water provided ad libitum in a 12-hour light/12-hour dark cycle at a room temperature of 21°C and humidity controlled at 43%. Experiments were performed using 8-10-week-old females. Male mice were used for mating from fertile age up until 1 year of age.

### Husbandry and timed pregnancies

Mating of 8–10-week-old females was initiated at 1 to 3 p.m. for five consecutive days. One male was paired with two females. Presence of a vaginal plug 7 to 9 a.m. the following morning was designated as gestational day (E) 0.5. Pregnancy was confirmed on E10.5 by a 10–15% body weight increase relative to E0.5. Fetal loss rate was calculated as the percentage of abortions among implantation sites.

### Fluorescence activated cell sorting for in vitro assays

To sort rare MMc cells and fetal hematopoietic stem and progenitor cells (fHSPC) from the bulk offspring’s cells, fetal BM cells of one litter was stained with fluorochrome-conjugated antibodies against extracellular antigens under sterile conditions (Supplementary Table 3). Immediately before sorting, cells were re-filtered in PBS-based buffer containing 2 mM EDTA to avoid cell clotting during sorting. Cell viability staining was performed by addition of 7-Aminoactinomycin D (7-AAD). Cells were sorted using an Aria Fusion flow sorter (BD). After sorting, >90% purity of the sorted populations was determined by flow cytometry. Per pooled litter (6–10 pups), on average 419 ± 72 (mean ± SEM) MMc and 1151 ± 224 (mean ± SEM) fHSPC could be obtained. Cells were sorted into PBS-based buffer containing 2 mM EDTA for subsequent co-culture.

### In vitro co-culture of MMc and fHSPC

For co-culture of sorted MMc and fHSPC (E18.5, derived from allogeneic mating between female C57BL/6 and male Balb/c), commercially available OP9 cells served as feeder layer. Confluent OP9 cells (described in ref. ^68^) were kept in αMEM medium (supplemented with 20% FCS (consistent lot number for all experiments) and 1% Penicillin/Streptomycin) under standard cell culture conditions (37°C, 5% CO_2_). Using this OP9 culture condition, differentiation towards the lymphoid lineage is low, since OP9 do not express Notch ligand and Delta-like 1 to promote T-cell differentiation^68,69^. Further, the culture was not supplemented with Flt-3L to enhance B-cell differentiation^70^. OP9 cells have been authenticated under RRID: CVCL_4398. Sorted MMc and fHSPC were added in a ratio of 1 MMc to 10 fHSPC cells onto a confluent OP9 feeder cell layer. This ratio corresponded to the previously determined in vivo ratio of fHSPC and MMc quantities, i.e., on average 1500 fHSPC and 100–150 MMc per million fetal cells in the fetal bone marrow on E18.5. fHSPC cells and MMc cells cultured alone served as control conditions. Cells were kept at 37°C, 5% CO_2_ for 120 h (5 days) without changing the medium. On culture day 5, sorted CD45^+^ cells were spun onto a glass slide and stained according to the Pappenheim protocol (Giemsa-May-Grünwald), followed by the morphological classification of cells based on standardized criteria for bone marrow cells. Here, early progenitors were defined by the structure and maturation of the nuclei, ratio of cytoplasmic to nuclear region, and additional classification criteria, as described in refs. ^71,72^. Scoring was performed at multiple randomized optical fields per sample.

Simultaneously, sorted CD45^+^ cells were also assessed by flow cytometry upon 5 days of culture. MMc cells cultured alone for 5 days remained present in the culture without expanding. All co-culture samples were exclusively gated for fetal differentiated cells, while MMc cells were excluded during gating in downstream flow cytometric analyses.

### General mouse experimental design

For pregnancy experiments, female mice (non-litter mates) of both groups were mated and housed in the same area in the animal facility to control for environmental influences on pregnancy progression. Experiments were independently repeated at least three times. Data from individual fetuses derived from at least 3 separate litters were used for analysis.

### Tissue collection and processing

To harvest fetal tissues, fetuses were sacrificed by decapitation on E18.5. Some pregnant mice were injected i.v. with 12.5 µl CD45.2-APC-Cy7 in 87.5 µl PBS 15 min prior to culling in order to exclude that maternal blood contamination during fetal tissue preparation may have confounded the planned assessments of MMc in fetal organs. In order to avoid differences in fetal size as confounders of the respective analyses, female offspring were exclusively used from litters with at least 7, but no more that 9 offspring in total. Fetal age differences were minimized by the strictly timed pregnancies and harmonized time of tissue harvest at 7:30 a.m. Individual tissues (bone marrow from femur and tibia, liver, spleen, thymus) were mechanically disrupted and filtered through a cell strainer to obtain single-cell suspensions. Erythrocyte lysis was required for spleen (pooled per litter to obtain sufficient cell counts) and liver samples. We deliberately chose to evaluate offspring at E18.5 and not at birth, since hematopoiesis occurs before birth and we aimed to exclude potential confounding effects altering offspring’s immune responses, e.g., upon microbial colonization commencing at birth. To harvest adult tissues, maternal peripheral blood was collected by retroorbital puncture in an EDTA-treated microvette. To generate single cell suspensions, thymus, lymph nodes, liver and spleen were grinded and washed through a cell strainer. Spleen and liver suspensions were subjected to erythrocyte lysis. Bone marrow from femur and tibia was flushed from the bone cavity followed by erythrocyte lysis.

### Magnetic-activated cell sorting-based pre-enrichment of MMc

In order to reduce the signal to noise ratio during flow cytometric acquisitions, the population of MMc was enriched upon isolation from fetal bone marrow, following protocols established for cell subsets with low frequencies^73^. Here, all cells harvested from fetal front and hind leg bones were incubated for 30 min at 4°C in normal rat serum and anti-mouse CD16/32 (TruStain fcX, Biolegend) to block unspecific FcγRII/III binding. The unwanted fetal cells were stained with APC-conjugated anti-H-2D^d^ antibody (diluted 1:200) for 30 min at 4°C, followed by incubation with magnetically labeled anti-APC MicroBeads (Miltenyi, Cat.-No.: 130-090-855), according to the manufacturer’s protocol. During the separation run through the magnetic-activated cell sorting (MACS) column, the unlabeled target cells (H-2D^b^-positive MMc) were collected in the flow-through fraction, while the unwanted fetal H-2D^d^ cells were retained within the column and later eluted after removal of the column from the separator (Supplementary Fig. 1a).

### Flow cytometry

Prior to antibody staining, cells were incubated in normal rat serum and anti-mouse CD16/32 (TruStain fcX, Biolegend) to block unspecific FcγRII/III binding. Fluorochrome-conjugated antibodies against extra- and intracellular antigens are summarized in Supplementary Table 3. Antibodies in pre-determined dilutions (1:50, 1:100, 1:200, or 1:400) were added and incubated for 30 min at 4°C on ice in the dark. Subsequently, cell viability staining was performed for 30 min at 4°C on ice in the dark. MMc were determined among fetal bone marrow cells based on a two-step approach. First, by the expression of CD45.2 and the absence of CD45.1. A second MMc refining gating step was performed on CD45.2^+^ cells by gating on the maternal strain-specific MHC class-I marker H-2D^b^ and the absence of H-2D^d^. See also gating strategy used to determine MMc upon MACS-based MMc-pre-enrichment, as described in Supplementary Fig. 1a. MMc subsets were characterized after the second step, based on respective lineage marker. Samples were acquired using a LSR Fortessa flow cytometer (BD), using 1 × 10^6^ cells per bone marrow sample and 0.2 × 10^6^ cells for the other fetal organs for MMc quantification. Absolute numbers of MMc were calculated based on their relative abundance among acquired fetal cells. Data were analyzed using FlowJo software (v9 and X). Doublet cells and dead cells were excluded from the analysis. Fluorescence minus one (FMO) controls, which contain all antibodies of a staining panel except one, were included as controls to set gating boundaries for cell populations positive for the respective missing antibody.

### IFN-γ inducibility in monocytes

Fetal bone marrow cells were isolated as described above and 0.5 × 10^6^ cells in 1 ml RPMI medium (10% FBS, 1% L-Glutamine, 1% Pen/Strep) were incubated for 4 h in 5 ml FACS tubes in the presence of ionomycin/phorbol myristate acetate (PMA) (I/P) (50 ng/ml). After 30 min of incubation, Brefeldin A (10 µg/ml) was added to each sample. Preparation for flow cytometry for IFN-γ expression in monocytes was performed as described above- IFN-γ inducibility in monocytes was determined by the fold change of INF-γ positive monocytes upon I/P stimulation over unstimulated monocytes.

### Adoptive cell transfer in *Rag2*^−/−^γ*c*^−/−^ pregnant females in order to generate MMc^low+AT^ offspring

In order to restore circulating adaptive immune cells in mothers, adaptive-immune-cell-deficient *Rag2*^−/−^γ*c*^−/−^ pregnant females (mated to Balb/c) were anesthetized using CO_2_ on E12.5 and retroorbitally adoptively transferred with 10 × 10^6^ cells isolated from peripheral blood, uterus-draining inguinal and paraaortic lymph nodes, and spleen of gestational-age matched C57BL/6 females (mated to Balb/c).

### MeDIP-Seq

MeDIP (Methylation Dependent ImmunoPrecipitation) was performed as recently published^74^, where enriched methylated DNA regions were identified by short read sequencing. Briefly, HSC were sorted from bone marrow of 10–11-week-old MMc^+^ and MMc^low^ offspring, following the gating strategy depicted in Supplementary Fig. 4. Genomic DNA was extracted from 1.5 × 10^5^ HSC upon resuspending the cells in 300 µl lysis buffer (7.5% NaHCO_3_) by phenol–chloroform extraction and EtOH precipitation. Isolated genomic DNA was fragmented by sonification (Bioruptor, Diagenode, 25 cycles (30 s on, 30 s off)) to an average fragment size of 50–100 bp. For quality control, 0.025 ng DNA from in vitro methylated pCR2.1 plasmid were added to each sample as a spike-in control. DNA was denatured at 98°C for 10 min and cooled on ice for 10 min. From each sample, 1/10 of the total amount was used an input control. For MeDIP pull-down, 5 µg of a 5-mc-specific antibody (Diagenode) were added to the rest of the sample and rotated for 2 h at 4°C. Subsequently, 50 µl M-280 anti-mouse IgG Dynabeads were added and the complexes were incubated on a rotation wheel for 2 h at 4°C. The following steps were performed in parallel with the input samples. RNA digestion was performed at 37°C for 30 min using 1.3 µl RNAse A (20–40 mg/ml) prior to elution and protein digestion at 55°C for 30 min using 200 µl elution buffer (50 mM Tris-HCl, 10 mM EDTA and 1% SDS), 4 µl proteinase K (40 mg/ml) and 7 µl CaCl_2_ (300 mM). DNA was extracted using phenol–chloroform extraction and EtOH precipitation as described above.

MeDIP-compatible next-generation sequencing libraries were generated using 5 ng DNA and the DNA SMART ChIP-Seq Kit (TaKaRa, Clontech, Cat-No.: 634865) suitable for single-stranded DNA. MeDIP libraries were sequenced on a NextSeq 500 system (Illumina, Cat-No.: 20024906) using single read (1 × 75) flow cells and 30 million reads per MeDIP sample and 10 million reads per Input control.

After quality control of MeDIP-seq data which is performed using FASTQC^75^, reads were aligned to the MM10 genome using BWA^76^, resulting in BAM files. The alignment files were analyzed using the IGV software. For the detection of differentially methylated regions (DMRs) and gene ontology analyses, the tool diffreps^77^ was utilized. Default parameters were used except for the adaption of the window size to 800 nt. The genes with significantly DMRs (*P* value <0.0001, G-test) were filtered and analyzed in IGV. Differentially methylated genes that were significantly upregulated (hypermethylated, log2FC > 1, *p*-adj. <0.05) or downregulated (hypomethylated, log2FC < -1, *p*-adj. <0.05) were categorized according to their biological function using WebGestalt (Supplementary Fig. 6c). Further analyses were supported by sam2bedgff.pl, deepTools bamCoverage, and circus software (see code availability).

### Murine cytomegalovirus infection

In order to restore circulating antibodies in mothers prior to neonatal viral infection and exclude an effect of maternal antibody deficiency on neonatal immunity, adaptive-immune-cell-deficient Rag2^-/-^γc^-/-^ pregnant females on E12.5 were subcutaneously injected with 200 μl of serum isolated from wild-type C57BL/6 females. Murine cytomegalovirus (MCMV, 3DR mutant) and preparation of MCMV stocks were performed following published protocols^78–80^.

Neonatal mice were infected within their first 24 h of life. Lung infections were performed by inoculations of a volume of 10 μl by probing the laryngopharynx with a pipette and extension of the neck (laryngopharyngeal infection). Viral load was determined by luciferase activity measurements. Explanted organs were kept in PBS, homogenized with TissueLyser II (Qiagen), centrifuged, and supernatants were measured for luciferase expression after the addition of native Coelenterazine with Lumat LB 9507 (Berthold Technologies). For lung, salivary glands, bone marrow, and liver, 1:10 dilutions were performed for measurements. The transplacental transfer of maternal anti-MCMV antibodies to the fetus confounding the course of the neonatal MCMV infection could be excluded, since anti-MCMV antibodies was undetectable in serum samples of selected WT and *Rag2*^*−/−*^γ*c*^*−/−*^ dams. Serum from MCMV-infected adult WT females taken 3 weeks post infection was used as positive control (Supplementary Fig. 5o). Non-pregnant *Rag2*^*−/−*^γ*c*^*−/−*^ mice housed long-term in the animal facility were used as sentinels. Analysis was outsourced to Biomedical Diagnostics (Hannover, Germany).

### In vitro MCMV plaque reduction assay

Monocytes were isolated from wild-type neonatal (postnatal day 0) bone marrow by MACS-based magnetic separation using a monocyte isolation kit (Miltenyi, Cat.-No.: 130-100-629). Cells were processed according to the manufacturer’s protocol except for the blocking step, which was adjusted by using 30 µl of blocking solution and an incubation time of 15 min before proceeding with the next step. A total of 40,000 bone marrow fibroblast cells (M2-10B4) alone or together with the neonatal monocytes in a 1:1 ratio were infected with MCMV in a multiplicity of infection (MOI) of 0.001, resuspended in culture media (RPMI 1640 with 10% FCS, 5% penicillin/streptomycin, L-Glutamine, 1 mM sodium pyruvate and 0.05 mM 2-Mercaptoethanol), and cultured as triplicates in 96-well plates at 37°C and 5% CO_2_. Three hours after infection a layer of methylcellulose was added to interfere with non-cell-contact-dependent virus spread. As determined by MCMV-encoded Gaussia luciferase activity, bone marrow-derived monocytes were not overtly permissive to MCMV infection and their presence in co-culture together did not significantly affect virus loads in the M2-10B4 cells (Supplementary Fig. 5p). Importantly, the viability of bone marrow-derived monocytes rapidly decreased when cells were kept in mono-culture, which made it impossible to maintain mono-cultures over a duration of more than 24 h. However, in co-culture, a significant number of these cells remained viable and could be traced. Here, we hardly detected mCherry^+^ MCMV-infected bone marrow-derived monocytes after exposure to a comparatively high MOI of 0.1. We found approx. 20-fold less mCherry^+^ MCMV-infected bone-marrow-derived monocytes (CD45^+^) compared to M2-10B4 cells (Supplementary Fig. 5p). The absolute numbers of plaques per well were acquired and mCherry expression of ten randomly chosen plaques per well were imaged for further data analysis. All plaques were analyzed at 4 days post infection. The infection rate of monocytes in this setting is less than 5% of M2-10B4. All images were processed in ImageJ and plaques manually demarcated for calculation of plaque size in µm^2^ resulting in 30 values per experiment.

### Placental and bone marrow histology

Placentae and whole femur (E18.5) were fixed in 4% formaldehyde solution, embedded in paraffin, and cut at the mid-sagittal plane into sections of 4 µm thickness. For placental histomorphological assessment, Masson-Goldner trichrome staining was performed. Bone marrow was stained with Sirius Red staining. Images were acquired using a slide scanner. Areas of junctional zone (JZ) and labyrinth (L) zone were quantified using the open-source software Pannoramic Viewer and an L/JZ ratio was calculated.

### Quantitative real-time PCR

For RT-qPCRs, RNA from fetal bone marrow was isolated according to the protocol with the RNeasy Plus Universal Minikit (Qiagen, Cat-No.: 73404), and cDNA synthesized (25°C 10 min, 50°C 10 min, 85°C 5 min 4°C storage) using SuperScript IV Vilo (Thermo Fisher Scientific, Cat.-No.: 11756050). Each sample was run in triplicates with a concentration of 100 ng/well cDNA. All used assays are commercially available, containing primer–probe mixes for *Gapdh* (Thermo Fisher Scientific, Cat.-No.: Mm99999915_g1), *b-Actin* (Thermo Fisher Scientific, Cat.-No.: Mm00607939_S1), *Il2 receptor-gamma-chain* (Thermo Fisher Scientific, Cat.-No.: Mm00434256_m1), *Rag2* (Thermo Fisher Scientific, Cat.-No.: Mm00501300_m1). Assays were run on QuantStudio 5 (Applied Biosystems). *Gapdh* and *b-Actin* served as internal control. Quantification of target mRNA relative to *Gapdh* and *b-Actin* was done using the comparative (ΔΔ) CT method.

### Microbiome analysis

Degenerate primers that contain the Illumina adapter consensus sequence F: (5′TCGTCGGCAGCGTCAGATGTGT ATAAGAGCAGCCTACGGGNGGCWGCAG-3′) and R: (5′GTCTCGTGGGCTCGGAG ATGTGTATAAGAGACAGGACTACHVGGGTATCTAATCC-3′) were used to generate V3–V4 region 16 S rRNA amplicons, following published protocols^81,82^. Samples were multiplexed using the Illumina Nextera XT Index Kit (Illumina, Cat-No.: FC-131-1001) to construct barcoded libraries, which were sequenced by 500PE sequencing on the MiSeq platform (Illumina, Cat-No.: MS-102-2003). The average quality scores of each sample before and after paired reads was determined using FastQC (Babraham Bioinformatics, Babraham Institute, UK). The paired ends in each sample were joined. All sequences less than 250 bp and/or with a Phred score <33 were excluded. QIIME 53 was used for quality filtering at Phred ≥ Q20. QIIME version 1.7 was used for operational taxonomic unit (OTU) clustering and alpha- and beta-diversity analysis^83^. USEARCH 8.1 was used as a chimera filter. All sequences were clustered based on 97% similarity to reference sequences. Reads not meeting these similarity criteria were clustered de novo. Based on the SILVA database, taxonomy levels of representative sequences were assigned at 95% similarity. Alpha diversity was calculated according to the Shannon diversity index. To determine if differences between the distributions of microbiota profiles from the respective datasets reached levels of significance, beta diversity analysis of similarities was performed.

### PRINCE study

The PRINCE study is a population-based prospective pregnancy study based at the University Medical Center Hamburg-Eppendorf and was initiated in 2011. Inclusion criteria were maternal age of 18 years or higher and a viable singleton pregnancy at gestational week 12–14. Women with chronic infections (HIV, Hepatitis B/C), known substance abuse, smoking, or pregnancies conceived after assisted reproductive technologies were excluded from study participation. Assessment of relevant covariables has been described in detail elsewhere^84^. All study participants signed informed consent forms and the study protocol was approved by the ethics committee of the Hamburg Chamber of Physicians under the registration number PV3694. For the present analyses, mother–child pairs have been identified among the PRINCE participants based on the following criteria: availability of maternal and cord blood samples and parental-reported information on early life respiratory infections of the child. The information on infections early in life was obtained via questionnaires filled by the parents and hence, identification of the pathogen causing the infection (viral or bacterial) was not included in the study design. See also demographic Supplementary Table 1.

### Cord blood processing

Cord blood was obtained from neonates born to participating women who gave birth at the University Medical Center Hamburg. Samples were processed within 2 h after delivery. Cord blood mononuclear cells (CBMC) were extracted from whole blood using Biocoll (Biochrome/Merck) gradient centrifugation and stored until analysis in liquid nitrogen in RPMI1640/20% heat-inactivated FBS supplemented with 10% DMSO. Maternal whole blood and fetal cord blood was frozen for subsequent DNA extraction.

### Microchimerism analysis in cord blood by DIP-specific duplex digital PCR

To screen for maternal microchimerism in cord blood, we adapted technical approaches developed to quantify hematopoietic chimerism after bone marrow transplantation (for details see supplementary information and ref. ^40^). Cryopreserved full blood samples from mother and cord blood were used to isolate genomic DNA, which was diluted to 50 ng per reaction. Specific primers and probes, their sequences and related screening kits are described in Supplementary data 3. In total, the presence or absence of 36 DIPs were tested by PCR, using hematopoietic cell kinase (HCK) as reference gene. Once informative DIPs were identified, CBMC stored in liquid nitrogen were thawed and the number of retrieved cells was counted. At least 5 × 10^6^ of CBMC were used for DNA isolation. Digital PCR was carried out using the QX100 Droplet Digital PCR System (Bio-Rad, Laboratories, USA). Up to 70 ng of CBMC-derived DNA could be isolated per cord blood sample using the QIAamp DNA Mini Kit (Qiagen, Cat-No.: 51306). To increase the chance of detecting MMc in cord blood, five reactions, each run in duplicates, of CBMC-derived DNA were performed using the maximum volume allowed by ddPCR Supermix (9.4 µl) (BIORAD, Cat-No.: 1863024). In addition, DNA isolated from maternal blood was used as positive control. The PCR mix was compartmentalized using the QX100 Droplet Generator. The produced droplet-containing plate was sealed and amplified using dPCR. Generated data was analyzed with the QuantaSoft software (Bio-Rad).

### Quantification and statistical analysis

Statistical parameters including sample size *n*, the definition of center, dispersion, and precision measures (mean ± SEM) and statistical significance are reported in the figures and figure legends. Data was tested for normal distribution and the hypothesis judged to be non-zero with statistical significance when *p* < 0.05 by Mann–Whitney-U test, Kruskal–Wallis test, or Wilcoxon-Rank-Sum test. In figures, asterisks denote statistical significance (*p < 0.05; **p < 0.01; ***p < 0.001; ****p < 0.0001). Human data analysis was conducted using nonparametric Spearman correlations with a 95% confidence interval. Outliers were removed by using Robust regression and Outlier removal function in GraphPad PRISM with a false discovery rate of 1%. Statistical analysis was performed in GraphPad PRISM 8.

### Reporting summary

Further information on research design is available in the Nature Research Reporting Summary linked to this article.

## Supplementary information

Supplementary Information

Description of Additional Supplementary Files

Supplementary Data 1

Supplementary Data 2

Supplementary Data 3

Reporting Summary

## Data Availability

Data sets generated within this study have been submitted to SRA (microbiome: NCBI Project with primary accession code SRP318008); or to GEO (methylome: NCBI GEO with primary accession code GSE151725); or to flowrepository.org (flow cytometry: Repository ID: FR-FCM-Z2ML). Additionally, for Fig. 5 and Supplementary Fig. 7, a supplementary data file (Supplementary data 3) contains the gene targets and primer sequences. For Supplementary Fig. 6, two supplementary data files (Supplementary data 1, 2) provide the raw methylome data. Further information and resources and reagents are available from the corresponding author on request. Source data are provided with this paper.

## Source data

Source Data

## Code availability

All codes used to analyze data in this study are available at https://github.com/huang/MMcPaper.
