# Supplementary material for: Vertically transferred maternal immune cells promote neonatal immunity against early life infections
Source: Nat Commun. 2021 Aug 4;12:4706. doi: 10.1038/s41467-021-24719-z (PMC8338998; doi:10.1038/s41467-021-24719-z)
Supplement: Supplementary file 1 — Supplementary Information [file 41467_2021_24719_MOESM1_ESM.pdf]

SUPPLEMENTAL INFORMATION

Vertically transferred maternal immune cells promote neonatal immunity against early life infections

Ina Annelies Stelzer[1,§,#], Christopher Urbschat[1,§], Steven Schepanski[1,2], Kristin Thiele[1], Ioanna Triviai[3], Agnes Wieczorek[1], Malik Alawi[4], Denise Ohnezeit[5], Julian Kottlau[7], Jiabin Huang[5], Nicole Fischer[5], Hans-Willi Mittrücker[6], Maria Emilia Solano[1], Boris Fehse[3], Anke Diemert[1], Felix R. Stahl[7], Petra Clara Arck[1,*]

[1]Division of Experimental Feto-Maternal Medicine, Department of Obstetrics and Fetal Medicine, University Medical Center Hamburg, 20246 Hamburg, Germany

[2]Developmental Neurophysiology, Center for Molecular Neurobiology Hamburg (ZMNH), University Medical Center Hamburg-Eppendorf, 20246 Hamburg, Germany

[3]Department of Stem Cell Transplantation, University Medical Center Hamburg-Eppendorf, 20246 Hamburg, Germany

[4]Bioinformatics Core, University Medical Center Hamburg-Eppendorf, Hamburg, Germany

[5]Institute for Medical Microbiology, Virology and Hygiene, University Medical Center Hamburg-Eppendorf, 20246 Hamburg, Germany

[6]Department of Immunology, University Medical Center Hamburg-Eppendorf, 20246 Hamburg, Germany

[7]Institute of Clinical Chemistry and Laboratory Medicine, University Medical Center Hamburg-Eppendorf, 20246 Hamburg, Germany

[§]Authors contributed equally.

[#]current affiliation: Department of Anesthesiology, Perioperative and Pain Medicine, Stanford University, Palo Alto 94305, California, USA

[*]Lead contact, Correspondence: Petra Arck p.arck@uke.de

This supplemental material includes:
Supplementary figures 1-7
Supplementary tables 1-3
Supplementary data 1-3

Supplementary Figure 1

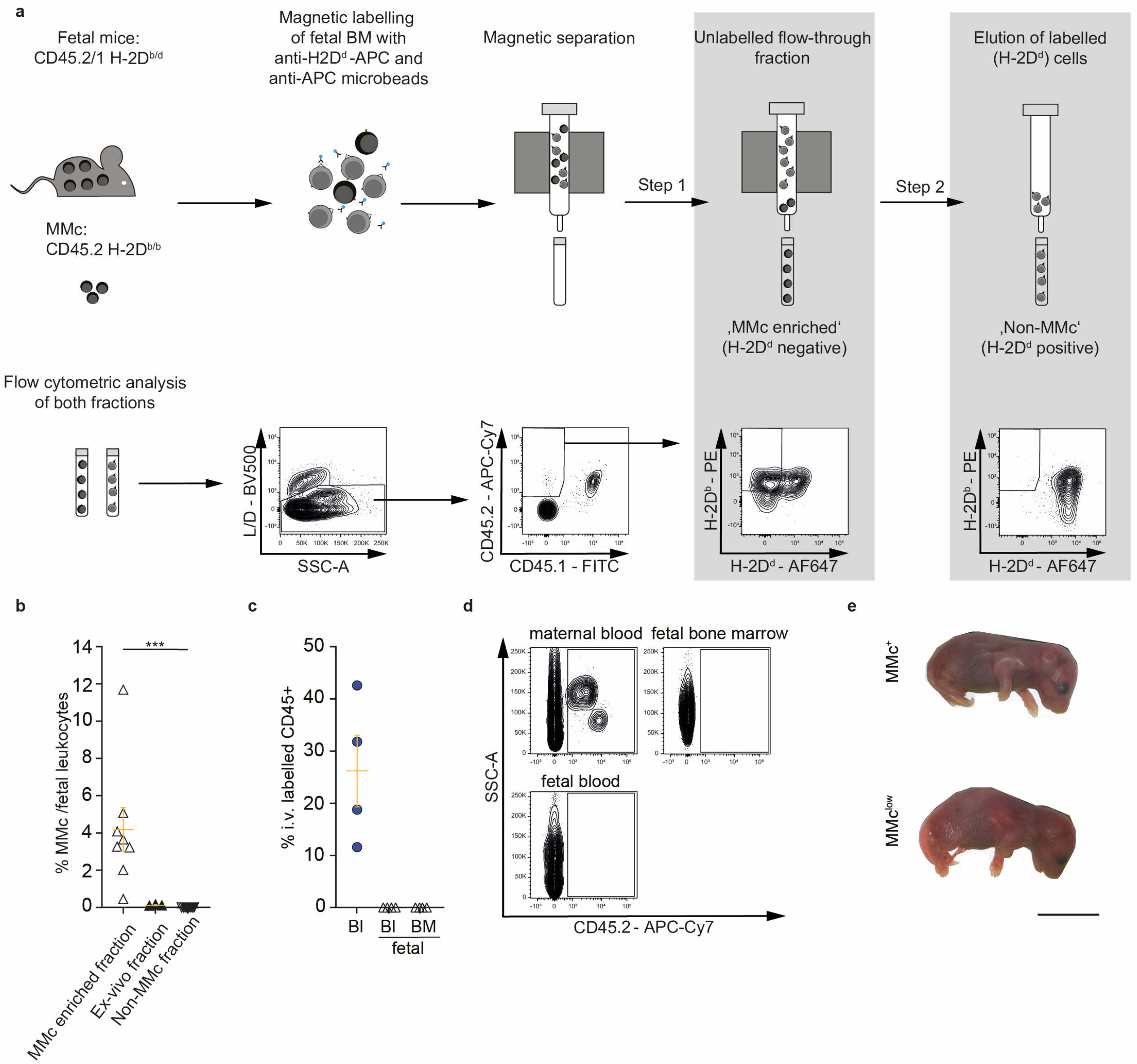

Supplementary Figure 1, related to Figure 1. Gating strategy for detection of maternal microchimeric cells by flow cytometry (after MACS enrichment) and evidence for contamination-free detection of maternal cells in fetal tissues.

a Strategy used to enrich MMc by magnetic-activated cell sorting prior to flow cytometry. Dot plots depict gating strategy to identify CD45.2 H-2D$^{b/b}$-positive MMc among fetal bone marrow (BM) expressing CD45.1/2 H-2$^{d/b}$. **b** Frequencies of MMc among fetal BM derived leukocytes on E18.5 upon MACS-enrichment, compared to non-MACS enriched *ex vivo* fraction. MMc enriched fraction n=8, *ex vivo* fraction n=3, scatter-plots show mean ± SEM, Kruskal-Wallis test, Dunn test for post-hoc analysis, two-sided, p=0.0003. **c** Percentage of labelled CD45$^+$ cells in maternal blood (Bl), fetal blood and BM after i.v. injection of anti-CD45 antibody prior to culling the pregnant mouse, all n=4. **d** Representative dot plots of data shown in (**c**). **e** Photograph of fetuses on E18.5 upon isolation from uterus, showing no signs of maternal blood contamination that might have confounded MMc detection in fetal tissues. Bar represents 1cm. Source data are provided as a Source Data file.

Supplementary Figure 2

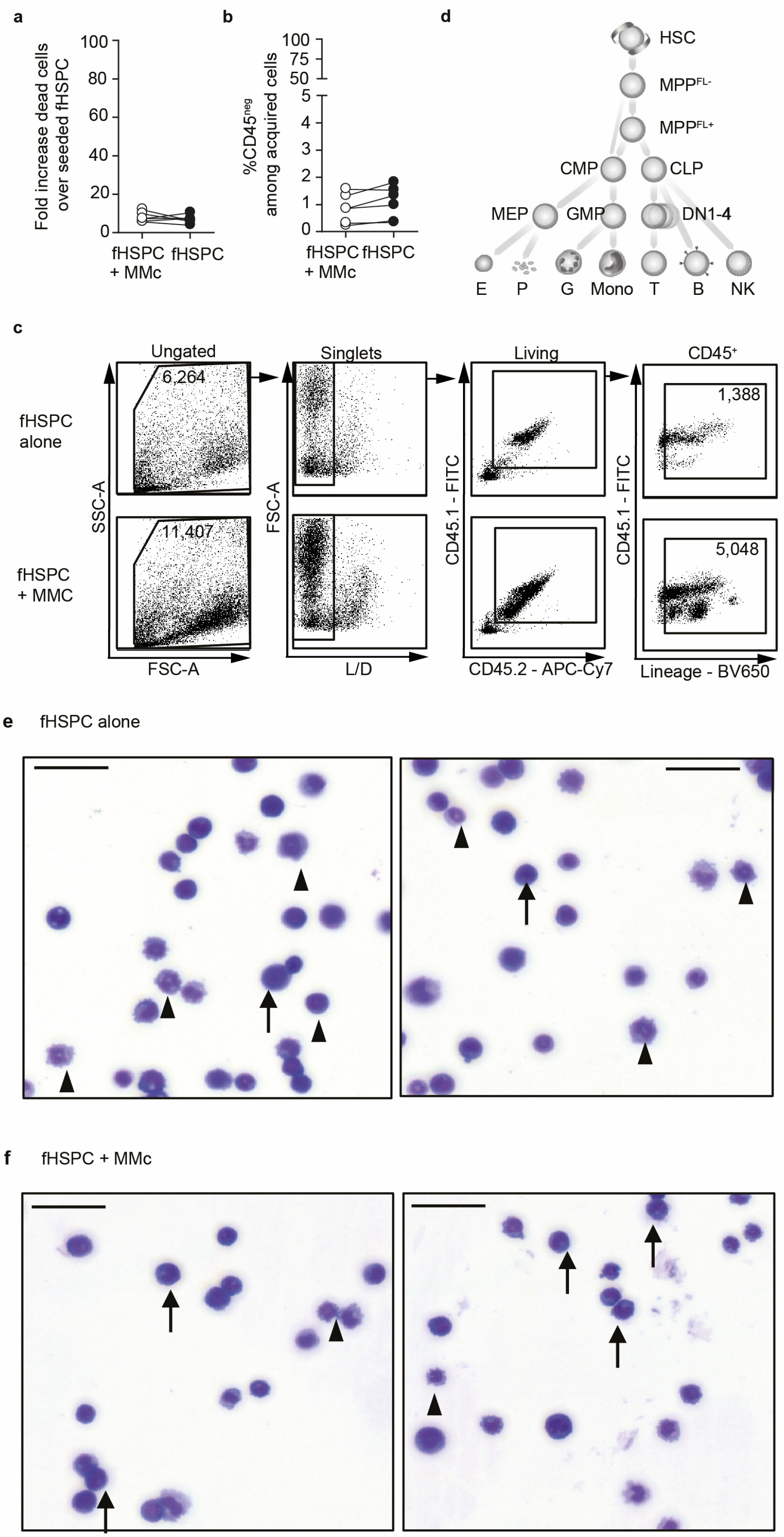

Supplementary Figure 2, related to Figure 1. MMc promote differentiation of fetal hematopoietic stem and progenitor cells (fHSPC) towards monocytes *in vitro*.

Flow cytometry-based assessment of (**a**) dead cell counts and (**b**) $CD45^{neg}$ stromal cell frequencies after 5 days of fetal hematopietic stem and progenitor cell (fHSPC) culture alone or in presence of MMc. Cells sorted and pooled from single litters (n=6), examined in independent experiments. **c** Gating strategy used to identify $CD45^{+}Lin^{+}$ cells differentiated from fHSPC 120hrs after (co-)culture initiation. **d** Simplified hematopoiesis diagram depicting the development of different blood cells from fHSPC to mature cells. **e, f** Representative cytospins of lineage-committed cells derived from fHSPC after 5 days of culture in presence or absence of MMC. Arrows: monocytic cell, arrow heads: granulocytic cell; cells pooled and sorted from single litters (n=8), examined in independent experiments. Bar=50µm. MPP: multipotent progenitor ($Flt3^{-}$, $Flt3^{+}$), CMP: common-myeloid progenitor, CLP: common-lymphoid progenitor, MEP: megakaryocyte-erythrocyte progenitor, GMP: granulocyte-monocyte progenitor, DN1-4: double-negative lymphocytes, stages 1-4, E: erythrocyte, P: platelet, G: granulocyte, Mono: monocyte, T: T cell, B: B cell, NK: natural killer cell. Source data are provided as a Source Data file.

Supplementary Figure 3

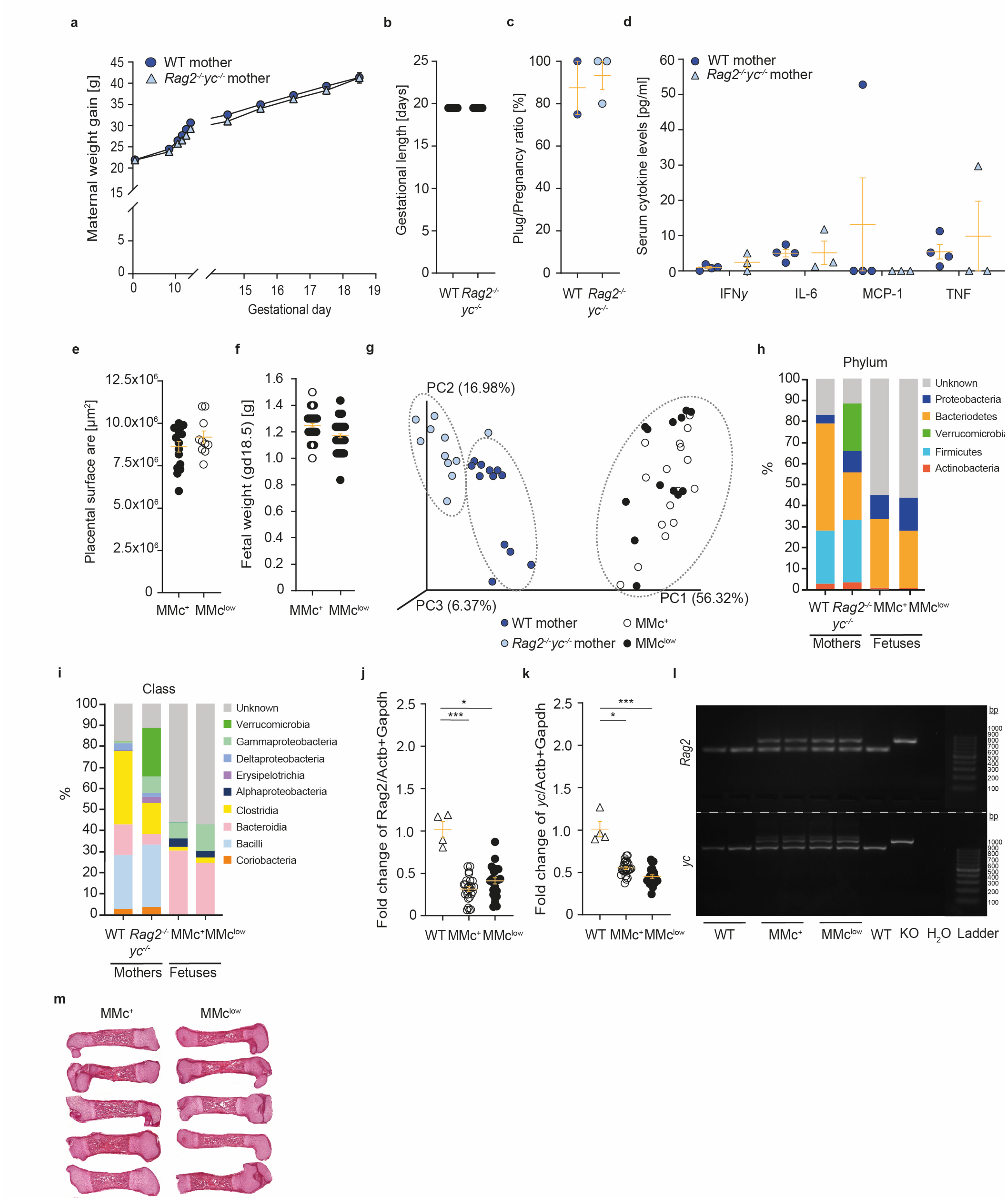

Supplementary Figure 3, related to Figure 2. Maternal genotype does not affect gestation, reproductive outcome, offspring's microbial diversity, and bone morphology.

a Maternal weight gain, **b** gestational length, **c** plug-to-pregnancy rate over the course of the experiments, **d** maternal serum cytokine levels, **e** placental surface area, **f** fetal weight (18.5) in MMc^{+} and MMc^{low} offspring. Number of animals: **a-c** WT n=16, *Rag2*$^{-/-}$*γc*$^{-/-}$ n=15, **d** WT n=4, *Rag2*$^{-/-}$*γc*$^{-/-}$ n=3, **e** WT n=16, *Rag2*$^{-/-}$*γc*$^{-/-}$ n=10, **f** MMc^{+} n=136, MMc^{low} n=95. Scatter-

plots represent mean ± SEM, mixed-effects analysis, t-test or Mann-Whitney-U test, two-sided. **g** Phylogenetic distances between intestinal microbiome samples, calculated via weighted UniFrac distance matrix. Each dot represents one microbiome sample, collected on postnatal day from mothers and neonates (n=8 each). Data between WT and *Rag2*$^{-/-}$*γc*$^{-/-}$ and respective offspring are depicted as weighted UniFrac distances, based on ANOSIM (p= 0.001) analysis. WT n=5, *Rag2*$^{-/-}$*γc*$^{-/-}$ n=4, MMc^{+} n=16, MMc^{low} n=13. Statistics are described in methods part. Relative abundances at the phylum (**h**) and class (**i**) level within the taxonomic composition of the gut microbiome in mothers and offspring. **j, k** Relative mRNA expression of *Rag2* and *yc* in MMc^{+}, MMc^{low} and WT offspring. *Rag2*: WT vs. MMc^{+}p=0.0006, WT vs. MMc^{low} p=0.0151. *yc:* WT vs. MMc^{+} p=0.033, WT vs. MMc^{low} p=0.0001, mean ± SEM, Kruskal-Wallis test, Dunn test for post-hoc analysis, two-sided. Number of animals: WT n=4, MMc^{+} n=26, MMc^{low} n=25. **l** Representative genotyping showing that MMc^{+} and MMc^{low} offspring have the same genotype (*Rag2*$^{+/-}$*γc*$^{+/}$), DNA isolated from n=6 females per group from n=6 independent litters. Base pair (bp) ladder indicates DNA amplicon length. **m** Histomorphology of fetal femurs on E18.5 from MMc^{+} and MMc^{low} offspring. Bar=500µm. Source data are provided as a Source Data file.

Supplementary Figure 4

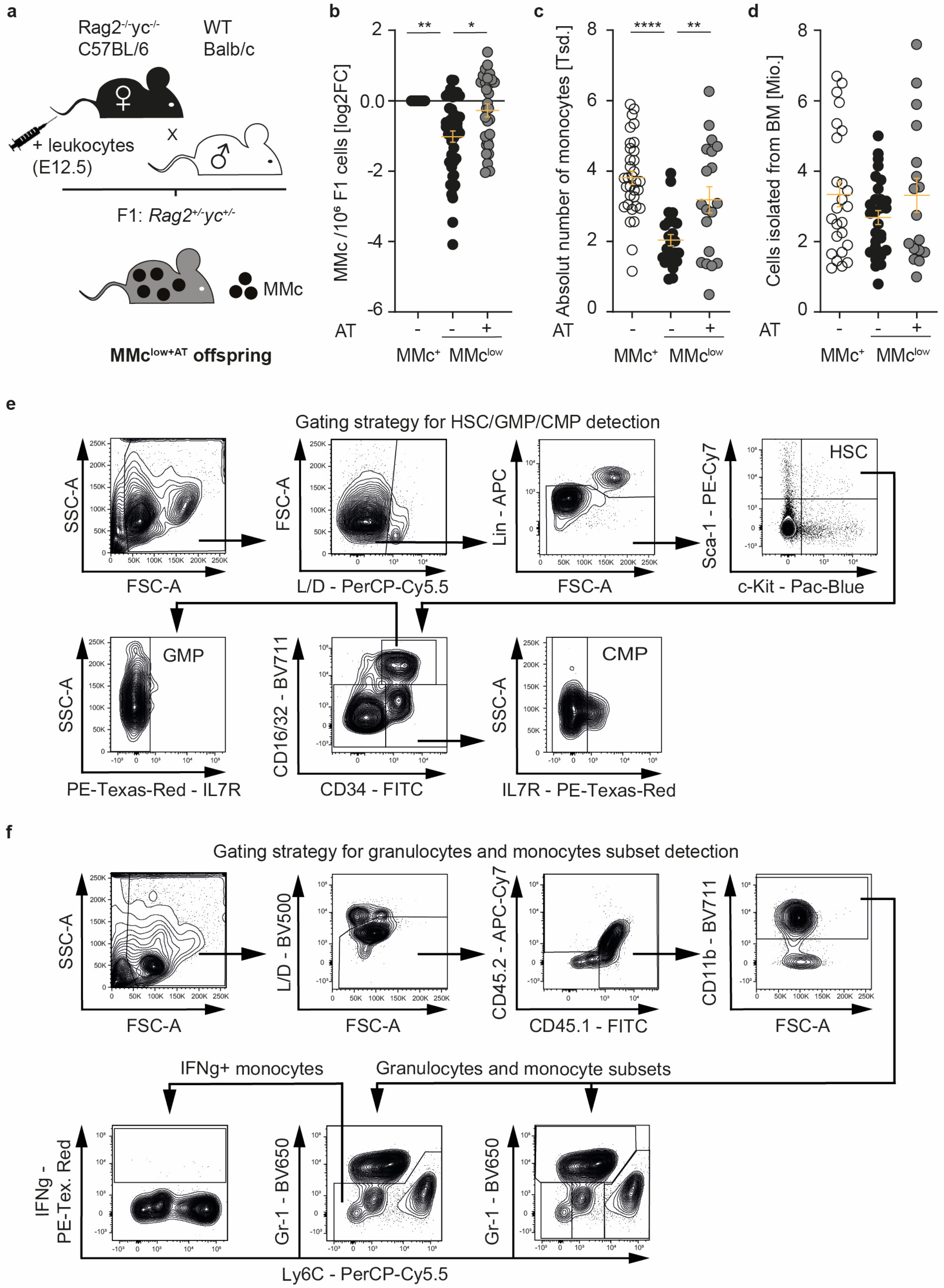

Supplementary Figure 4, related to Figure 3. The number of MMc in MMc^{low} offspring can be restored by adoptive transfer of immune cells in *$Rag2^{-/-}\gamma c^{-/-}$* mothers during mid-gestation.

a Additional mating set up designed to restore the MMc transfer during pregnancy in *$Rag2^{-/-}\gamma c^{-/-}$* mothers by adoptive transfer (AT) of immune cells. Resulting offspring were termed MMc^{low+AT}. **b** Number of MMc /1 x 10^6 fetal cells in fetal bone marrow on E18.5 in MMc^{low} and MMc^{low+AT}, relative to the number in MMc^+ offspring (log2FC). MMc^+ n=18, MMc^{low} n=46, and MMc^{low+AT} n=29. Kruskal-Wallis test, Dunn test for post-hoc analysis, two-sided, MMc^+ vs. MMc^{low} p=0.0023; MMc^{low} vs. MMc^{low+AT} p=0.0112. **c** Absolute numbers of monocytes (x 1000; Tsd.) recorded by flow cytometry, MMc^+ n=33, MMc^{low} n=24, MMc^{low+AT} n=19. One-Way ANOVA, Tukey-test for post-hoc analysis, two-sided, MMc^+ vs. MMc^{low} p=0.0001, MMc^{low} vs. MMc^{low+AT} p=0.0058. **d** Absolute numbers of cells (x 10^6; Mio.) isolated from fetal bone marrow, MMc^+ n=33, MMc^{low} n=24, MMc^{low+AT} n=19. **b-d** Scatter-plots represent mean ± SEM. **e** Gating strategy used for detection of HSC (defined as Lin^- $Sca\text{-}1^+$ $c\text{-}Kit^+$), CMP (defined as $Sca\text{-}1^{neg}$, $c\text{-}Kit^+$, $CD34^+$, $CD16/32^{neg}$, $IL\text{-}7R^+$), GMP (defined as $Sca\text{-}1^{neg}$, $c\text{-}Kit^+$, $CD34^+$, $CD16/32^+$, $IL\text{-}7R^{neg}$) and CLP (defined as $Sca\text{-}1^{neg}$, $c\text{-}Kit^{neg}$, $IL7R^+$) in fetal bone marrow (E18.5). **f** Gating strategy for myeloid subsets in the fetal bone marrow on E18.5. Source data are provided as a Source Data file.

Supplementary Figure 5

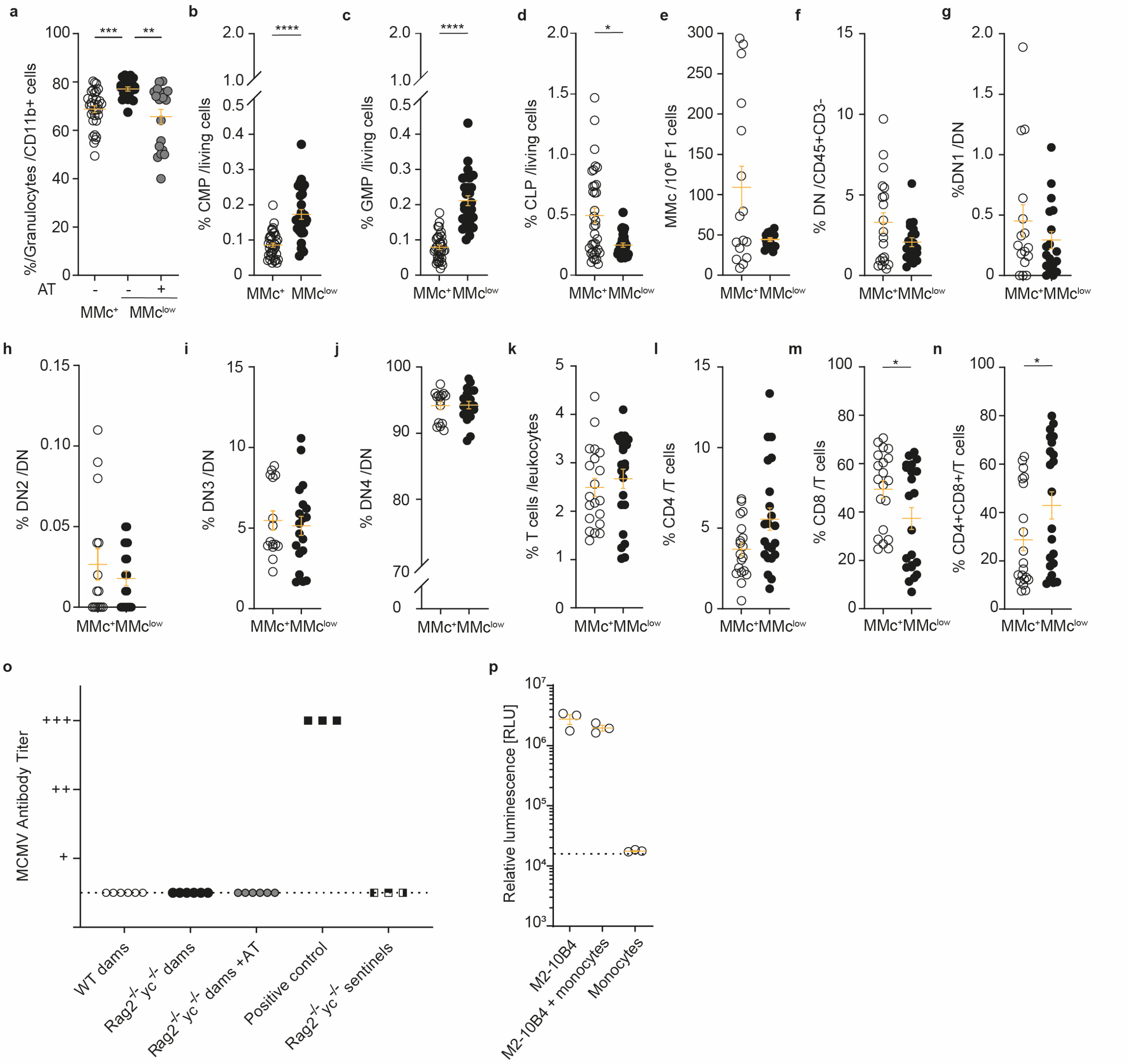

Supplementary Figure 5, related to Figure 3+4. Differentiation of the myeloid lineage in the fetal bone marrow and the lymphoid lineage in the thymus is affected by maternal microchimeric cells.

In fetal bone marrow of female MMc^+ n=33 offspring vs. MMc^{low} n=23 and MMc^{low+AT} n=19: **a** frequencies of granulocytes among $CD11b^+$ myeloid cells, MMc^+ vs. MMc^{low} *p*=0.0002, MMc^{low} vs. MMc^{low+AT} *p*=0.0020; **b** CMP, MMc^+ vs MMc^{low} *p*=0.0001; **c** GMP, MMc^+ vs MMc^{low} *p*=0.0001; **d** CLP, MMc^+ vs MMc^{low} *p*=0.0132. Among fetal thymic cells at E18.5, n=17 MMc^+, n=17 MMc^{low} offspring: **e** MMc/1 x 10^6; **f-n** frequencies of T cell progenitors (double-negative (DN)) and terminally differentiated $CD4^+$ and $CD8^+$ single-positive T cells. MMc^+ n=20, MMc^{low} n=22. Scatter-plots represent mean ± SEM, Mann-Whitney-U test, Dunn test for post-hoc analysis, two-sided. **m** MMc^+ vs. MMc^{low} *p*=0.0418. **n** MMc^+ vs. MMc^{low} *p*=0.0451. **o** Murine cytomegalovirus (MCMV) antibody titer analysis in serum of mothers from all three mating groups. WT dams n=6, *Rag2*$^{-/-}$*γc*$^{-/-}$ dams n=6*,* *Rag2*$^{-/-}$*γc*$^{-/-}$ dams +AT n=6, positive post-MCMV-infection control mother n=3, *Rag2*$^{-/-}$*γc*$^{-/-}$ sentinels n=3. **p** Viral loads of bone marrow fibroblasts in mono- or co-culture with monocytes, analysed by activity of MCMV-encoded *Gaussia* luciferase expression. Dotted line represents detection limit and was calculated from data obtained from non-infected cell cultures. Representative data from n=2 experiments are depicted. Source data are provided as a Source Data file.

Supplementary Figure 6

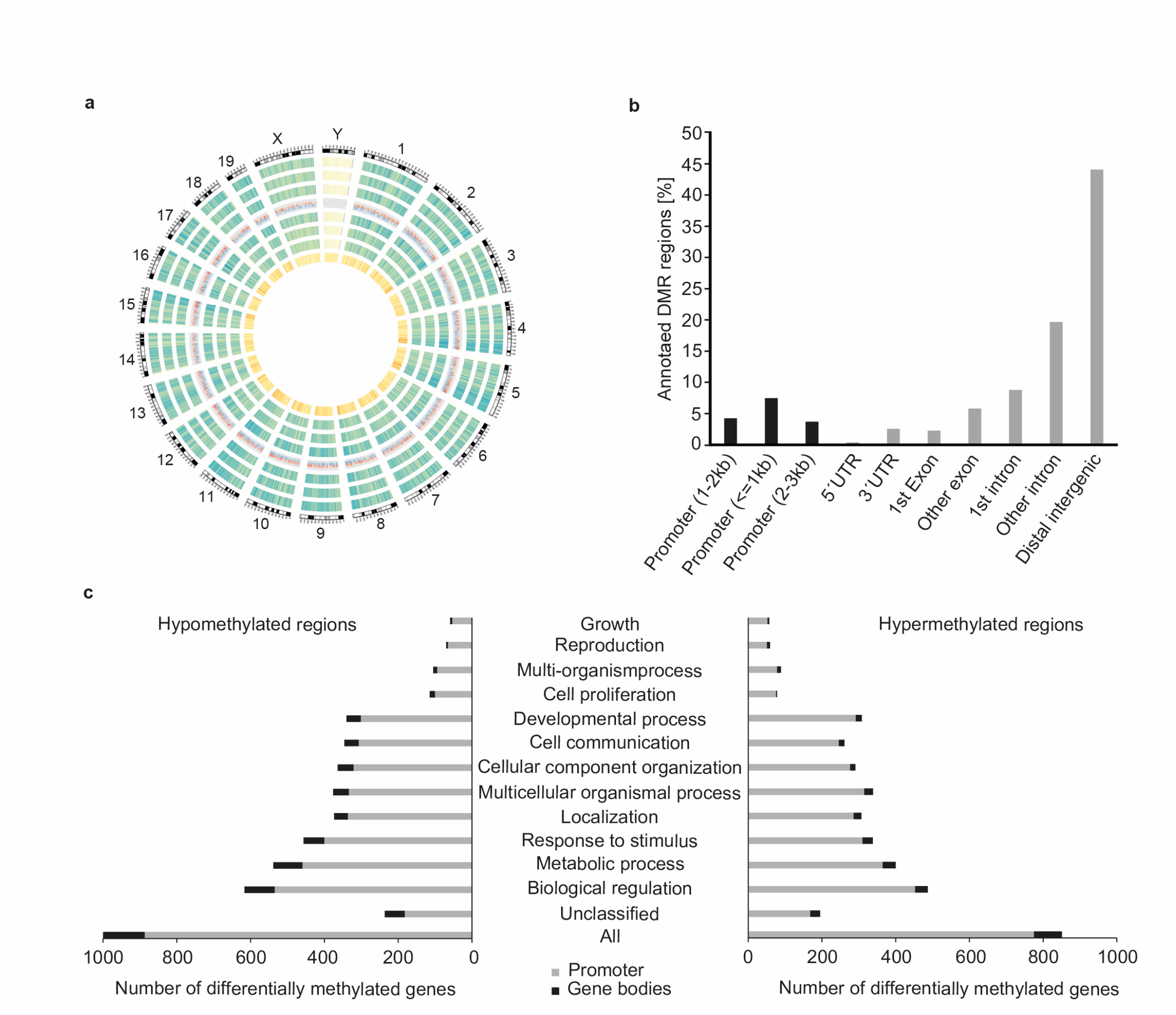

Supplementary Figure 6. Genome-wide methylation changes in HSCs from adult MMc^{+} and MMc^{low} offspring.

a Circos plot showing the genome-wide DNA methylation profile by chromosome in HSC from bone marrow of MMc^{+} and MMc^{low} offspring. The normalized short read coverages for each 100,000 bp non-overlapping bins are computed and displayed along with the significant (p-value < 0.0001) differentially methylated regions (DMRs). The outermost circle shows an ideogram of the mouse genome subdivided by chromosomes, followed by three outer circles showing the methylation levels in cells derived from MMc^{+} offspring. The middle circle depicts log2 fold changes of significant DMRs comparing HSCs from MMc^{low} vs. MMc^{+} mice. Red indicates hypermethylation in HSCs from MMc^{low} vs MMc^{+} mice, blue indicates hypomethylation. The three inner circles depict the methylation status of MMc^{low} offspring. Blue indicates high levels and yellow indicates low levels of methylation. The innermost circle represents CpG density. **b** Graph depicting the genomic distribution of DMRs of MMc^{low} vs. MMc^{+}. **c** Gene Ontology (GO) analysis with differentially methylated genes. **a-c** n=3 samples per group, each sample consists of biological replicates from a pool of n=3 animals, resulting in n=9 animals per group. Source data are provided as Supplementary data files 1, 2.

Supplementary Figure 7

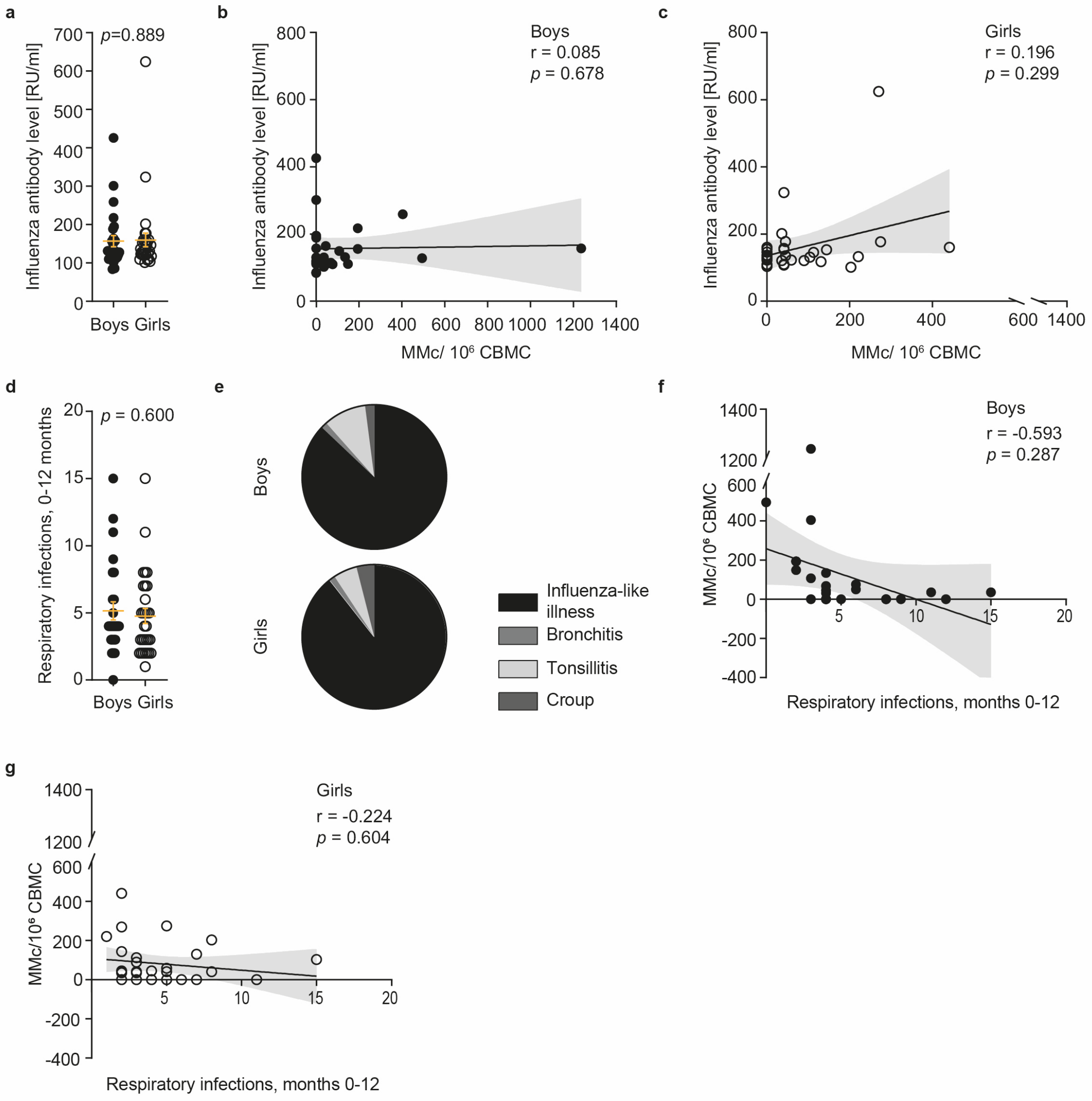

Supplementary Figure 7, related to Figure 5. Maternal microchimerism in cord blood and early life infections in human infants in their first year of life.

a Influenza antibody levels measured in neonatal serum obtained from cord blood at delivery. **b, c** Correlation of influenza antibody levels and number of MMc in cord blood mononuclear cells (CBMC) of boys and girls. **d** Questionnaire-based self-reported total number of respiratory infections in children during the first year of life. **e** Specified respiratory infections reported for boys and girls in their first year of life. **f, g** Correlation of number of MMc and respiratory infections in the first year of life in boys and girls. **a, d** Graphs represent mean ± SEM and Mann-Whitney U test. **b, c, f, g** Linear regression line (black), nonparametric Spearman correlation with a 95% confidence interval (shaded). Additional data are provided in supplementary data file 3.

Supplementary table 1: Sociodemographic data of children participating in the PRINCE study and included in the analysis

		PRINCE cohort 0-6 months (mean ± SD)	PRINCE cohort 7-12 months (mean ± SD)
**Gestational age (weeks)**			
	Girls	38.8 ± 1.5	38.6 ± 1.6
	Boys	39.2 ± 1.1	39.2 ± 1.2
**Birth weight (grams)**			
	Girls	3480.1 ± 435.2	3408.7 ± 435.4
	Boys	3567.6 ± 488.8	3534.2 ± 520.7
**Birth height (cm)**			
	Girls	51.5 ± 2.0	51.3 ± 2.1
	Boys	52.5 ± 2.5	52.5 ± 2.7
**Siblings (count)**			
	Girls	0.4 ± 0.5	0.4 ± 0.5
	Boys	0.2 ± 0.4	0.2 ± 0.4

Supplementary table 2: Multiple regression analysis of infection risk assessment in children during the first year of life (0-12 months) and second half of the first year of life (7-12 months)

					95% CI		
		n	β	*p* value	lower	upper	r
			**7-12 months**				
**MMc/ 10^6 CBMC**							
	Girls	30	-0.008	0.221	-0.020	0.005	-0.032
	Boys	26	-0.004	0.031	-0.007	-0.0004	-0.416
**Influenza antibody level (RU/ml)**							
	Girls	30	0.013	0.077	-0.001	0.027	-0.051
	Boys	26	0.011	0.062	-0.001	0.023	0.193
**Gestational age**							
	Girls	30	-0.446	0.298	-1.313	0.421	-0.271
	Boys	26	0.239	0.646	-0.829	1.308	0.199
**Birth weight**							
	Girls	30	-0.001	0.379	-0.004	0.002	-0.342
	Boys	26	-0.001	0.454	-0.003	0.001	0.073
**Older siblings**							
	Girls	30	-0.234	0.852	-2.804	2.335	0.103
	Boys	26	0.038	0.971	-2.112	2.189	-0.142
			**0-12 months**				
**MMc/ 10^6 CBMC**							
	Girls	30	-0.003	0.604	-0.016	0.010	-0.224
	Boys	26	-0.003	0.287	-0.010	0.003	-0.593
**Influenza antibody level (RU/ml)**							
	Girls	30	-0.006	0.428	-0.021	0.009	-0.278
	Boys	26	-0.005	0.592	-0.025	0.015	-0.206
**Gestational age**							
	Girls	30	-0.087	0.853	-1.042	0.868	-0.093
	Boys	26	-0.584	0.508	-2.392	1.223	-0.421
**Birth weight**							
	Girls	30	-0.001	0.534	-0.004	0.002	-0.131
	Boys	26	-0.001	0.538	-0.005	0.003	-0.106
**Older siblings**							
	Girls	30	0.061	0.534	-2.848	2.970	-0.013
	Boys	26	-0.864	0.651	-4.787	3.060	0.180

CBMC: Cord blood mononuclear cells, CI: confidence interval, RU: relative units. P value estimates the significance of a relationship between variables by an F-test, meaning the slope is not equal zero.

Supplementary table 3, rel. to Figure 1, 2, 4. Antibodies used in flow cytometry and MeDIP immunoprecipitation

Antigen/Target	Conjugated fluorochrome	Clone	Company	Dilution	Catalog number	Identifier
**CD45.2**	APC-Cy7	104	Biolegend	1:100	109824	RRID:AB_830789
**CD45.1**	FITC	A20	Biolegend	1:400	110706	RRID:AB_313495
**H-2Db**	PE	KH95	Biolegend	1:20	111508	RRID:AB_313513
**H-2Dd**	Alexa Fluor 647	34-2-12	Biolegend	1:200	110612	RRID:AB_492913
**CD3**	PE eFluor®610	145-2C11	eBioscience	1:200	61-0031-80	RRID:AB_2574513
**CD3**	BV421	145-2C11	Biolegend	1:100	100335	RRID:AB_10898314
**CD3**	PerCP-Cy5.5	145-2C11	eBioscience	1:100	45-0031-82	RRID:AB_1107000
**CD3**	PE-Cy7	145-2C11	Biolegend	1:200	100320	RRID:AB_312685
**B220**	BV 650	RA3-6B2	Biolegend	1:100	103241	RRID:AB_11204069
**CD11c**	BV 785	N418	Biolegend	1:100	117335	RRID:AB_11219204
**CD11b**	PE-Cy7	M1/70	Biolegend	1:400	101216	RRID:AB_312799
**CD11b**	BV711	M1/70	Biolegend	1:200	101241	RRID:AB_11218791
**Ly-6C**	PerCP-Cy5.5	HK1.4	eBioscience	1:200	45-5932-82	RRID:AB_2723343
**Gr-1**	BV570	RB6-8C5	Biolegend		108431	RRID:AB_10896783
**Gr-1**	BV650	RB6-8C5	Biolegend	1:200	108441	RRID:AB_2562401
**IFNγ**	PE-CF594	XMG1.2	Becton Dickinson	1:200	562333	RRID:AB_11154588
**Lineage cocktail**	Biotinylated	CD5, CD11b, B220, Anti-7-4, Anti-Ly6-G/C, TER-119	Miltenyi Biotec	1:400	130-092-613	RRID:AB_1103214
**Lineage cocktail**	APC	CD3 (145-2C11), Ly-6G/C (RB6-8C5), CD11b (M1/70), B220 (RA3-6B2), Ly-76 (Ter-119)	Becton Dickinson	1:400	51-9003632	Not authenticated by RRID
**Sca-1 (Ly-6A/E)**	BV711	D7	Biolegend	1:100	108131	RRID:AB_493596
**Sca-1 (Ly-6A/E)**	PE-Cy7	D7	Biolegend	1:100	108114	RRID:AB_493596
**c-Kit (CD117)**	BV421	2B8	Biolegend	1:100	105828	RRID:AB_11204256
**c-Kit (CD117)**	BV605	ACK2	Biolegend	1:100	135121	RRID:AB_2562040
**IL-7R (CD127)**	PE-CF594	SB/199	Becton Dickinson	1:200	562419	RRID:AB_11153131
**CD34**	FITC	RAM34	Becton Dickinson	1:200	553733	RRID:AB_395017
**CD135**	PE	A2F10.1	Becton Dickinson	1:100	553842	RRID:AB_395079
**CD48**	APC-Cy7	HM48-1	Biolegend	1:100	103431	RRID:AB_2561462
**CD45**	BV510	30-F11	Biolegend	1:200	103137	RRID:AB_2561392
**CD150 (SLAM)**	BV785	TC15-12F12.2	Biolegend	1:100	115937	RRID:AB_2565962
**CD16/32**	BV711	93	Biolegend	1:400	101337	RRID:AB_2565637
**Viability dye**	eFluor® 506	na	eBioscience	1:250	65-0866	Not authenticated by RRID
**7-AAD viability staining**	na	na	Biolegend	1:400	420404	Not authenticated by RRID
**Biotin**	Streptavidin-BV650	na	Biolegend	1:200	405231	Not authenticated by RRID
**5-methylcytosine**	Conjugated to OVA	33D3	Diagenode		C15200081-100	RRID:AB_2572207
**CD4**	FITC	RM4-5	Becton Dickinson	1:400	553047	RRID:AB_394583
**CD8**	Bv711	53-6.7	Biolegend	1:100	100759	RRID:AB_2563510
**CD45**	APC-Cy7	30-F11	Becton Dickinson	1:200	557659	RRID:AB_396774
