# Supplementary material for: Vertically transferred maternal immune cells promote neonatal immunity against early life infections
Source: Nat Commun. 2021 Aug 4;12:4706. doi: 10.1038/s41467-021-24719-z (PMC8338998; doi:10.1038/s41467-021-24719-z)
Supplement: Supplementary file 2 — Description of Additional Supplementary Files [file 41467_2021_24719_MOESM2_ESM.pdf]

Description of Additional Supplementary Files

File Name: Supplementary Data 1
Description: **related to Supplementary Figure 6a.xlsx**

Raw data of genome-wide DNA methylation profiles in hematopoietic stem cells (HSC) derived from MMc^{+} and MMc^{low} offspring mice by chromosome

File Name: Supplementary Data 2
Description: **related to Supplementary Figure 6b-c.xlsx**

Raw data of differentially methylated regions of HSC of adult MMc^{+} and MMc^{low} offspring.

File Name: Supplementary Data 3
Description: **related to Figure 5 and Supplementary Figure 7.xlsx**

Genes and primer sequences used to detect MMc in human cordblood
