# Supplementary material for: Vertically transferred maternal immune cells promote neonatal immunity against early life infections
Source: Nat Commun. 2021 Aug 4;12:4706. doi: 10.1038/s41467-021-24719-z (PMC8338998; doi:10.1038/s41467-021-24719-z)
Supplement: Supplementary file 6 — Reporting Summary [file 41467_2021_24719_MOESM6_ESM.pdf]

nature research

Corresponding author(s): Petra Arck

Last updated by author(s): May 5, 2021

Reporting Summary

Nature Research wishes to improve the reproducibility of the work that we publish. This form provides structure for consistency and transparency in reporting. For further information on Nature Research policies, see our Editorial Policies and the Editorial Policy Checklist.

Statistics

For all statistical analyses, confirm that the following items are present in the figure legend, table legend, main text, or Methods section.

n/a	Confirmed	
☐	☒	The exact sample size (n) for each experimental group/condition, given as a discrete number and unit of measurement
☐	☒	A statement on whether measurements were taken from distinct samples or whether the same sample was measured repeatedly
☐	☒	The statistical test(s) used AND whether they are one- or two-sided<br>*Only common tests should be described solely by name; describe more complex techniques in the Methods section.*
☒	☐	A description of all covariates tested
☐	☒	A description of any assumptions or corrections, such as tests of normality and adjustment for multiple comparisons
☐	☒	A full description of the statistical parameters including central tendency (e.g. means) or other basic estimates (e.g. regression coefficient) AND variation (e.g. standard deviation) or associated estimates of uncertainty (e.g. confidence intervals)
☐	☒	For null hypothesis testing, the test statistic (e.g. F, t, r) with confidence intervals, effect sizes, degrees of freedom and P value noted<br>*Give P values as exact values whenever suitable.*
☒	☐	For Bayesian analysis, information on the choice of priors and Markov chain Monte Carlo settings
☒	☐	For hierarchical and complex designs, identification of the appropriate level for tests and full reporting of outcomes
☐	☒	Estimates of effect sizes (e.g. Cohen's d, Pearson's r), indicating how they were calculated

Our web collection on statistics for biologists contains articles on many of the points above.

Software and code

Policy information about availability of computer code

Data collection	BD FACS DIVA v9, Becton Dickinson;
Data analysis	FlowJo, v9 and X. FLowJo LLC.; Prism v.8, GraphPad; QuantaSoft v1.7, BioRad; Pannoramic Viewer v1.15.4, 3D Histech; diffreps; fastqc; BWA; IGV; Code related to this study (sam2bedgff.pl; deepTools bamCoverage; circos) is available here: https://github.com/huang/MMcPaper

For manuscripts utilizing custom algorithms or software that are central to the research but not yet described in published literature, software must be made available to editors and reviewers. We strongly encourage code deposition in a community repository (e.g. GitHub). See the Nature Research guidelines for submitting code & software for further information.

Data

Policy information about availability of data

All manuscripts must include a data availability statement. This statement should provide the following information, where applicable:

- Accession codes, unique identifiers, or web links for publicly available datasets
- A list of figures that have associated raw data
- A description of any restrictions on data availability

Source data are provided with this paper. Datasets generated during this study and related to microbiome, methylome, and flow cytometry analyses of MMc are publicly available (microbiome: NCBI (https://www.ncbi.nlm.nih.gov/sra/?term=SRP318008) Project SRP318008, Accession numbers SRR14369765-SRR14369817; methylome: GEO (https://www.ncbi.nlm.nih.gov/geo/query/acc.cgi?acc=GSE151725), Accession numbers GSM4589122, GSM4589123, GSM4589124, GSM4589125, GSM4589126, GSM4589127, GSM4589128, GSM4589129, GSM4589130, GSM4589131, GSM4589132, GSM4589133, and ERP122069; flow cytometry: flowrepository.org, Repository ID: FR-FCM-Z2ML). For Supplementary Figure 6, two supplementary data files (Supplementary data 1, 2) provides the raw methylome data. For Figure 5, a supplementary data file (Supplementary data 3) contains the gene targets and primer sequences. Further information and requests for resources and reagents should be directed to and will be fulfilled by the Lead Contact.

Field-specific reporting

Please select the one below that is the best fit for your research. If you are not sure, read the appropriate sections before making your selection.

- [x] Life sciences
- [ ] Behavioural & social sciences
- [ ] Ecological, evolutionary & environmental sciences

For a reference copy of the document with all sections, see nature.com/documents/nr-reporting-summary-flat.pdf

Life sciences study design

All studies must disclose on these points even when the disclosure is negative.

Sample size	An a priori power analysis was conducted using G*Power v3.1 software (Heinrich Heine Universität Düsseldorf), anticipating a moderate effect size of 0.5. Since we expected a high interindividual variability for detecting rare cells, 7 pregnant female with their respective litters (mean litter size 7 pups) needed to be included to reach a power of 0.95.<br>Sample size of the human cohort was determined based on the following criteria: availability of paired maternal and cord blood samples, and parental-reported information on early life respiratory infections of the child. Among 127 dyads in which a maternal-specific DIP was detected, medical records on early life infections were available for 26 male and 30 female infants. Based on the high interindividual variability expected for detecting rare cells in the cordblood, this sample size was statistically sufficient to correlate the number of early life infections with the number of maternal cells.
Data exclusions	Outlier were excluded based on GraphPad Prism ROUT outlier analysis, Q = 1%.
Replication	All experiments were independently repeated at least three times, while all attempts at replication were successful.
Randomization	Control and treated/MMclow vs. MMcpos females were mated, treated, and measured in parallel in every independently repeated experiment. Pregnant female mice (Rag2gc-/-) were randomly split into +/- adoptive transfer groups.<br>For the analyses involving human samples samples were non-randomly allocated into two experimental groups, based on the sex of the neonate. To control for the influence of potential covariates, a confounder analysis was performed for both groups, confirming that the number of infections was not influenced by covariates other than the number of maternal cells present in the cordblood.
Blinding	Investigators were not blinded during the experiments or analysis of the data, as the experimental approaches did not involve randomization and patient treatment as part of a clinical study. Single experiments and analyses in animals were conducted in a blinded manner, where possible and feasible, to ensure objectivity.

Reporting for specific materials, systems and methods

We require information from authors about some types of materials, experimental systems and methods used in many studies. Here, indicate whether each material, system or method listed is relevant to your study. If you are not sure if a list item applies to your research, read the appropriate section before selecting a response.

Materials & experimental systems

n/a	Involved in the study	
[ ]	[x]	Antibodies
[ ]	[x]	Eukaryotic cell lines
[x]	[ ]	Palaeontology and archaeology
[ ]	[x]	Animals and other organisms
[ ]	[x]	Human research participants
[x]	[ ]	Clinical data
[x]	[ ]	Dual use research of concern

Methods

n/a	Involved in the study	
[ ]	[x]	ChIP-seq
[ ]	[x]	Flow cytometry
[x]	[ ]	MRI-based neuroimaging

Antibodies

Antibodies used

Antigen Conjugated fluorochrome Clone Company Catalog number Identifier
CD45.2 APC-Cy7 104 Biolegend 109824 RRID:AB_830789
CD45.1 FITC A20 Biolegend 110706 RRID:AB_313495
H-2Db PE KH95 Biolegend 111508 RRID:AB_313513
H-2Dd Alexa Fluor 647 34-2-12 Biolegend 110612 RRID:AB_492913
CD3 PE eFluor®610 145-2C11 eBioscience 61-0031-80 RRID:AB_2574513
CD3 BV421 145-2C11 Biolegend 100335 RRID:AB_10898314
B220 BV 650 RA3-6B2 Biolegend 103241 RRID:AB_11204069
CD11c BV 785 N418 Biolegend 117335 RRID:AB_11219204
CD11b PE-Cy7 M1/70 Biolegend 101216 RRID:AB_312799
Ly-6C PerCP-Cy5.5 HK1.4 eBioscience 45-5932-82 RRID:AB_2723343

Gr-1 BV570 RB6-8C5 Biolegend 108431 RRID:AB_10896783
Gr-1 BV650 RB6-8C5 Biolegend 108441 RRID:AB_2562401
IFNg PE-CF594 XMG1.2 Becton Dickinson 562333 RRID:AB_11154588
Lineage cocktail Biotinylated CD5, CD11b, B220, Anti-7-4, Anti-Ly6-G/C, TER-119 Miltenyi Biotec 130-092-613 RRID:AB_1103214
Lineage cocktail APC CD3 (145-2C11), Ly-6G/C (RB6-8C5), CD11b (M1/70), B220 (RA3-6B2), Ly-76 (Ter-119) Becton Dickinson 51-9003632 Not authenticated by RRID
Sca-1 (Ly-6A/E) BV711 D7 Biolegend 108114 RRID:AB_493596
c-Kit (CD117) BV421 2B8 Biolegend 105828 RRID:AB_11204256
IL-7R (CD127) PE-CF594 SB/199 Becton Dickinson 562419 RRID:AB_11153131
CD34 FITC RAM34 Becton Dickinson 553733 RRID:AB_395017
CD16/32 BV711 93 Biolegend 101337 RRID:AB_2565637
Viability dye eFluor® 506 na eBioscience 65-0866 Not authenticated by RRID
7-AAD viability staining PerCP-Cy5.5 na Biolegend 420404 Not authenticated by RRID
Biotin Streptavidin-BV650 na Biolegend 405231 Not authenticated by RRID
5-methylcytosine Conjugated to OVA 33D3 Diagenode C15200081-100 RRID:AB_2572207

Validation

All antibodies were validated by the respective manufacturer, as indicated in the specific data sheets. For antibodies against CD45.2, CD45.1, H-2Db, H-2Dd, CD3, B220, CD11c, CD11b, Gr-1, Sca-1, c-Kit, CD16/32, all supplied by Biolegend, each lot of antibody is quality control tested by immunofluorescent staining with flow cytometric analysis by the supplier. Antibodies against CD3, and Ly-6C, supplied by eBioscience, have been tested by flow cytometric analysis of mouse splenocytes by the supplier. For the antibody against IFN-g, supplied by Becton Dickinson, the antibody is routinely quality-control tested for intracellular staining (flow cytometry) by the supplier. For Lineage antibody cocktail, and antibodies IL-7R, and CD34, supplied by Becton Dickinson, the antibodies are routinely quality-control tested for flow cytometry by the supplier. For Lineage antibody cocktail, supplied by Miltenyi Biotec, mouse bone marrow cells were test-stained with the cocktail and analyzed by flow cytometry by the supplier. For the antibody against 5-methylcytosine, the supplier Diagenode provides validation data for its application in MeDIP on its homepage.
For quality control, we titrate each antibody to determine its dilution for optimal performance for each application, and repeat its titration after receipt of each new lot.

Eukaryotic cell lines

Policy information about cell lines

Cell line source(s)

OP9 (ATCC CRL-2749), M2-10B4 (ATCC CRL-1972)

Authentication

The OP9 cell line is authenticated under RRID: CVCL_4398. We did not re-authenticate the cell line. The M2-10B4 cell line is authenticated under RRID:CVCL_5794. We did not re-authenticate this cell line.

Mycoplasma contamination

Both cell lines were tested for mycoplasma contamination, and found to be negative.

Commonly misidentified lines
(See ICLAC register)

We did not use any commonly misidentified cell lines in this study.

Animals and other organisms

Policy information about studies involving animals; ARRIVE guidelines recommended for reporting animal research

Laboratory animals

C57BL/6J, C57BL/6 Rag2-/-Il2rg-/-, and Balb/c Rag2-/-Il2rg-/- mice were obtained from the animal breeding facility of University Medical Center Hamburg-Eppendorf. Balb/c CD45.1 (CByJ.SJL(B6)-Ptprca/J) were purchased from The Jackson Laboratory. Mice were single-housed (males) or maintained in groups (females) in the animal facility of University Medical Center Hamburg-Eppendorf with regular chow and water provided ad libitum in a normal 12-hour light/12-hour dark cycle at a room temperature of 21°C and controlled humidity at 43%. Experiments were performed using 8-10-week-old females. Males were used for mating from fertile age up until 1 year of age.

Wild animals

Study did not involve wild animals.

Field-collected samples

Study did not involve samples collected in the field.

Ethics oversight

Animal care and all experimental procedures were performed according to University Medical Center Hamburg-Eppendorf institutional guidelines and conform to requirements of the German Animal Welfare Act. Ethical approvals were obtained from the State Authority of Hamburg (Behörde für Gesundheit und Verbraucherschutz, Hansestadt Hamburg, Germany, approval numbers G10/067, G16/085, G17/010, G17/099, ORG_615, ORG_702, ORG_764, ORG_795).

Note that full information on the approval of the study protocol must also be provided in the manuscript.

Human research participants

Policy information about studies involving human research participants

Population characteristics

The PRINCE study is a population-based prospective pregnancy study based at the University Medical Center Hamburg-

	Eppendorf and was initiated in 2011. Inclusion criteria were maternal age of 18 years or higher and a viable singleton pregnancy at gestational week 12–14. Women with chronic infections (HIV, Hepatitis B/C), known substance abuse, smoking, pregnancies conceived after assisted reproductive technologies were excluded from study participation. Assessment of relevant covariables has been described in detail in Diemert et al., J Reprod Immunol 2017, PMID: 28641119.
Recruitment	The recruitment took place at the University Medical Center Hamburg-Eppendorf and in private practices in the city of Hamburg during routine prenatal care visits. A potential bias in the study population is the selection of women who are open to participate in scientific studies and are approached by their physician.
Ethics oversight	All study subjects signed informed consent forms and the study protocol was approved by the ethics committee of the Hamburg Chamber of Physicians under the registration number PV3694.

Note that full information on the approval of the study protocol must also be provided in the manuscript.

ChIP-seq

Data deposition

- [x] Confirm that both raw and final processed data have been deposited in a public database such as GEO.
- [x] Confirm that you have deposited or provided access to graph files (e.g. BED files) for the called peaks.

Data access links<br>*May remain private before publication.*	The MeDIP-Seq data analyzed in this study are publicly available through GEO or ENA under accessions GSM4589122, GSM4589123, GSM4589124, GSM4589125, GSM4589126, GSM4589127, GSM4589128, GSM4589129, GSM4589130, GSM4589131, GSM4589132, GSM4589133, and ERP122069
Files in database submission	MMC_2neg_R1.fastq.gz MMC_2pos_R1.fastq.gz MMC_4neg_R1.fastq.gz MMC_4pos_R1.fastq.gz MMC_5neg_R1.fastq.gz MMC_5pos_R1.fastq.gz MMC_2neg_input_R1.fastq.gz MMC_2pos_input_R1.fastq.gz MMC_4neg_input_R1.fastq.gz MMC_4pos_input_R1.fastq.gz MMC_5neg_input_R1.fastq.gz MMC_5pos_input_R1.fastq.gz MMC_2neg_rpkm.bw MMC_2pos_rpkm.bw MMC_4neg_rpkm.bw MMC_4pos_rpkm.bw MMC_5neg_rpkm.bw MMC_5pos_rpkm.bw MMC_2neg_input_rpkm.bw MMC_2pos_input_rpkm.bw MMC_4neg_input_rpkm.bw MMC_4pos_input_rpkm.bw MMC_5neg_input_rpkm.bw MMC_5pos_input_rpkm.bw 800.diff.gt.bed
Genome browser session<br>(e.g. UCSC)	Final_igv_session.xml

Methodology

Replicates	MeDIPs from DNA isolated from bone-marrow derived HPSCs from offspring mice; n=3
Sequencing depth	MMC_2neg: depth 2.42, 19489273 reads, 18965363 uniquely mapped reads, 84nt long, single-end<br>MMC_4neg: depth 2.35, 25283648 reads, 24625670 uniquely mapped reads, 84nt long, single-end<br>MMC_5neg: depth 2.45, 25314350 reads, 24645674 uniquely mapped reads, 84nt long, single-end<br>MMC_2pos: depth 2.22, 26241384 reads, 25031096 uniquely mapped reads, 84nt long, single-end<br>MMC_4pos: depth 2.52, 29500504 reads, 27620386 uniquely mapped reads, 84nt long, single-end<br>MMC_5pos: depth 2.59, 33070616 reads, 31880532 uniquely mapped reads, 84nt long, single-end
Antibodies	5-methylcytosine (5-mC) Antibody - clone 33D3 (Diagenode), C15200081-100, Lot Nr. GF005
Peak calling parameters	--nsd broad --gname mm10 --window 800 --frag 250
Data quality	--meth gt --pval 0.0001
Software	diffReps.pl

Flow Cytometry

Plots

Confirm that:

- [x] The axis labels state the marker and fluorochrome used (e.g. CD4-FITC).
- [x] The axis scales are clearly visible. Include numbers along axes only for bottom left plot of group (a 'group' is an analysis of identical markers).
- [x] All plots are contour plots with outliers or pseudocolor plots.
- [x] A numerical value for number of cells or percentage (with statistics) is provided.

Methodology

Sample preparation	Individual fetal and neonatal mouse tissues (bone marrow from femur and tibia, liver, spleen, thymus) were mechanically disrupted and filtered through a cell strainer to obtain single-cell suspensions. Erythrocyte lysis was required for spleen (pooled per litter to obtain sufficient cell counts) and liver samples. To harvest adult tissues, maternal peripheral blood was

	collected by retroorbital puncture in an EDTA-treated microvette. To generate single cell suspensions, thymus, lymph nodes and spleen were grinded and washed through a cell strainer. Spleen suspensions were subjected to erythrocyte lysis. Bone marrow from femur and tibia was flushed from the bone cavity followed by erythrocyte lysis.
Instrument	BD Fortessa II, BD Aria Fusion, BD Symphony A3
Software	FACS DIVA v9, BD; FlowJo v9 and X, FlowJo, LLC.
Cell population abundance	Purity of sorted populations was determined to be above 90%.
Gating strategy	Ungated events were first gated for all leukocytes based on forward- and sideward scatter (FSC/SSC) characteristics for size and granularity, respectively. Doublets were then excluded using (1) FSC-H vs. FSC-W, and (2) SSC-H vs. SSC-W. Next, viable (7-AAD- or Fixable Viability Dye-negative) cells were selected to gate living, singlet leukocytes that were used for downstream gating analyses as specified in the supplementary materials. Gate boundaries between positive and negative cell subsets were determined using fluorescence-minus-one (FMO) staining protocols and, where necessary, isotype-control stainings.

☒ Tick this box to confirm that a figure exemplifying the gating strategy is provided in the Supplementary Information.
